# Characterization of the First “*Candidatus* Nitrotoga” Isolate Reveals Metabolic Versatility and Separate Evolution of Widespread Nitrite-Oxidizing Bacteria

**DOI:** 10.1128/mBio.01186-18

**Published:** 2018-07-10

**Authors:** Katharina Kitzinger, Hanna Koch, Sebastian Lücker, Christopher J. Sedlacek, Craig Herbold, Jasmin Schwarz, Anne Daebeler, Anna J. Mueller, Michael Lukumbuzya, Stefano Romano, Nikolaus Leisch, Søren Michael Karst, Rasmus Kirkegaard, Mads Albertsen, Per Halkjær Nielsen, Michael Wagner, Holger Daims

**Affiliations:** a Division of Microbial Ecology, Department of Microbiology and Ecosystem Science, Research Network “Chemistry meets Microbiology,” University of Vienna, Vienna, Austria; b Max-Planck-Institute for Marine Microbiology, Bremen, Germany; c Department of Microbiology, Radboud University, Nijmegen, The Netherlands; d Center for Microbial Communities, Department of Chemistry and Bioscience, Aalborg University, Aalborg, Denmark; University of Southern California

**Keywords:** *Archaea*, Nitrotoga, activated sludge, ecophysiology, genome analysis, isolate, nitrification, nitrite oxidation

## Abstract

Nitrification is a key process of the biogeochemical nitrogen cycle and of biological wastewater treatment. The second step, nitrite oxidation to nitrate, is catalyzed by phylogenetically diverse, chemolithoautotrophic nitrite-oxidizing bacteria (NOB). Uncultured NOB from the genus “*Candidatus* Nitrotoga” are widespread in natural and engineered ecosystems. Knowledge about their biology is sparse, because no genomic information and no pure “*Ca*. Nitrotoga” culture was available. Here we obtained the first “*Ca*. Nitrotoga” isolate from activated sludge. This organism, “*Candidatus* Nitrotoga fabula,” prefers higher temperatures (>20°C; optimum, 24 to 28°C) than previous “*Ca*. Nitrotoga” enrichments, which were described as cold-adapted NOB. “*Ca*. Nitrotoga fabula” also showed an unusually high tolerance to nitrite (activity at 30 mM NO_2_^−^) and nitrate (up to 25 mM NO_3_^−^). Nitrite oxidation followed Michaelis-Menten kinetics, with an apparent *K*_*m*_ (*K*_*m*(app)_) of ~89 µM nitrite and a *V*_max_ of ~28 µmol of nitrite per mg of protein per h. Key metabolic pathways of “*Ca*. Nitrotoga fabula” were reconstructed from the closed genome. “*Ca*. Nitrotoga fabula” possesses a new type of periplasmic nitrite oxidoreductase belonging to a lineage of mostly uncharacterized proteins. This novel enzyme indicates (i) separate evolution of nitrite oxidation in “*Ca*. Nitrotoga” and other NOB, (ii) the possible existence of phylogenetically diverse, unrecognized NOB, and (iii) together with new metagenomic data, the potential existence of nitrite-oxidizing archaea. For carbon fixation, “*Ca*. Nitrotoga fabula” uses the Calvin-Benson-Bassham cycle. It also carries genes encoding complete pathways for hydrogen and sulfite oxidation, suggesting that alternative energy metabolisms enable “*Ca*. Nitrotoga fabula” to survive nitrite depletion and colonize new niches.

## IMPORTANCE

Nitrite-oxidizing bacteria (NOB) are major players in the biogeochemical nitrogen cycle and critical for wastewater treatment. However, most NOB remain uncultured, and their biology is poorly understood. Here, we obtained the first isolate from the environmentally widespread NOB genus “*Candidatus* Nitrotoga” and performed a detailed physiological and genomic characterization of this organism (“*Candidatus* Nitrotoga fabula”). Differences between key phenotypic properties of “*Ca*. Nitrotoga fabula” and those of previously enriched “*Ca*. Nitrotoga” members reveal an unexpectedly broad range of physiological adaptations in this genus. Moreover, genes encoding components of energy metabolisms outside nitrification suggest that “*Ca*. Nitrotoga” are ecologically more flexible than previously anticipated. The identification of a novel nitrite-oxidizing enzyme in “*Ca*. Nitrotoga fabula” expands our picture of the evolutionary history of nitrification and might lead to discoveries of novel nitrite oxidizers. Altogether, this study provides urgently needed insights into the biology of understudied but environmentally and biotechnologically important microorganisms.

## INTRODUCTION

Nitrification, the microbially catalyzed oxidation of ammonia via nitrite to nitrate, is a key process of the natural biogeochemical nitrogen cycle. Nitrification also is critical for the removal of excess nitrogen from sewage in wastewater treatment plants (WWTPs), whereas in agriculture, it contributes to the loss of nitrogen from fertilized soils (1). The first step of nitrification—ammonia oxidation to nitrite—is carried out by chemolithoautotrophic ammonia-oxidizing bacteria and archaea, whereas the second step—nitrite oxidation to nitrate—is catalyzed by chemolithoautotrophic nitrite-oxidizing bacteria (NOB).

NOB are the main biological source of nitrate, an important nitrogen source for many plants and microorganisms and an electron acceptor used by many microbes under anoxic conditions. Additionally, NOB have a strong impact on marine carbon cycling (2). Recently, surprising discoveries have been made in NOB-related research, demonstrating alternative energy metabolisms, such as the oxidation of hydrogen, sulfide, formate, and other organic compounds in organisms previously described as obligate nitrifiers (3–5). Furthermore, a novel “reciprocal feeding” interaction of NOB from the genus *Nitrospira* with ammonia oxidizers was described, where the NOB initiate nitrification by releasing ammonia from urea or cyanate (4, 6). Another surprise was the discovery of photolithoautotrophic NOB that use nitrite as an electron donor for anoxygenic photosynthesis (7) and most likely evolved independently of the chemolithoautotrophic NOB (8). For decades, a core paradigm of nitrification research stated that ammonia and nitrite oxidation are always catalyzed by distinct organisms, which cooperate by cross-feeding. This long-standing opinion was contradicted by the discovery of complete nitrifiers (comammox organisms) in the NOB genus *Nitrospira*, which perform both steps of nitrification (9, 10). All NOB known until recently belong to the classes *Alpha*- and *Gammaproteobacteria*, the phylum *Nitrospirae*, or the phylum *Nitrospinae* (11–13). The known phylogenetic diversity of NOB has been now expanded by the description of several new NOB lineages: the genus *Nitrolancea* in the *Chloroflexi* (14), the candidate genus “*Candidatus* Nitromaritima” in the *Nitrospinae* (15), and the candidate genus “*Candidatus* Nitrotoga” in the *Betaproteobacteria*, family *Gallionellaceae* (16).

Past research demonstrated that *Nitrospira* bacteria are the major NOB in many WWTPs (17, 18). However, “*Candidatus* Nitrotoga” has recently been recognized as another widely distributed and sometimes predominant group of NOB in WWTPs (19–21). Other known habitats of “*Ca*. Nitrotoga” include soil, sediment, tap water and recirculation aquaculture biofilms, caves, and subglacial lake ecosystems (16, 22–26). Despite their importance, little is known about the microbiology of “*Ca*. Nitrotoga.” The first representative, “*Ca*. Nitrotoga arctica,” was enriched from Siberian permafrost soil (16). This organism and “*Ca*. Nitrotoga” members enriched from activated sludge (21) or eelgrass sediment (22) are adapted to cold temperatures. Moreover, a slightly acidic pH (5.7 to 6.8) and elevated nitrite loading were reported to favor growth of “*Ca*. Nitrotoga” over *Nitrospira* (24, 25, 27). In addition, the kinetics of nitrite oxidation were studied using enriched “*Ca*. Nitrotoga” members (22, 28). Further characterization of “*Ca*. Nitrotoga,” including the nature of its nitrite-oxidizing enzyme and potential for alternative energy metabolisms, has been hampered by the lack of any pure culture or genome sequence from this genus.

In this study, we obtained the first “*Ca*. Nitrotoga” isolate, characterized its key physiological properties, and analyzed its genetic repertoire based on the fully sequenced genome. The new strain, which has been isolated from a municipal WWTP, shows remarkably different physiological adaptations than the previously described “*Ca*. Nitrotoga” enrichments. Phylogenetic analysis of its nitrite oxidoreductase (NXR), the key enzyme for nitrite oxidation, suggests that the evolutionary history of NOB is more complex than previously assumed and indicates that a surprising diversity of yet undiscovered bacterial and archaeal nitrite oxidizers may exist in nature.

## RESULTS AND DISCUSSION

### Isolation of a new “*Ca*. Nitrotoga” species.

After inoculation of mineral medium (3) containing nitrite with nitrifying activated sludge from a municipal wastewater treatment plant (WWTP) and repeated feeding with nitrite, a nitrite-oxidizing primary enrichment culture was obtained. An initial analysis of the culture by 16S rRNA-targeted fluorescence *in situ* hybridization (FISH) revealed the presence of *Nitrospira*, “*Ca*. Nitrotoga,” and other bacteria. Aliquots of this culture were regularly diluted in fresh nitrite-containing medium and incubated to further enrich the NOB. After the third dilution and transfer step, planktonic “*Ca*. Nitrotoga” cells were still detected by FISH in the culture, whereas *Nitrospira* cells were not found. *Nitrospira* might still have been present in abundances below the detection limit of FISH of approximately 10^4^ target cells per ml (29). The cause of the prevalence of “*Ca*. Nitrotoga” at this stage of enrichment remains unknown. In addition, this secondary enrichment contained other bacteria that were probably feeding on organic compounds produced by the autotrophic NOB.

**FIG 1:**
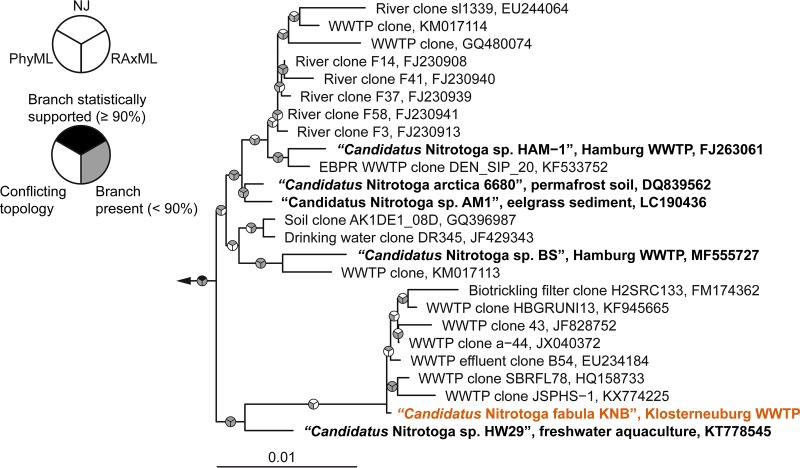
Phylogenetic affiliation of “*Candidatus* Nitrotoga fabula.” The consensus tree, which is based on 16S rRNA gene sequences of cultured and uncultured members of the candidate genus “Nitrotoga” shows the position of the “*Ca*. Nitrotoga fabula” isolate (indicated in orange) in this genus. Other cultured (enriched) “*Ca*. Nitrotoga” strains are indicated by boldface type. Pie charts indicate statistical support of branches based on maximum likelihood (RAxML; 1,000 bootstrap iterations) and neighbor joining (NJ) (1,000 bootstrap iterations). For PhyML, no bootstrap analysis was performed and gray indicates the presence of a branch. Bar, 0.01 estimated substitutions per nucleotide.

Since all further attempts to purify “*Ca*. Nitrotoga” in liquid culture were unsuccessful, the capability of this nitrite oxidizer to grow on solid medium containing nitrite was tested. Except for some *Nitrobacter* strains (30) and Nitrolancea hollandica (14), no pure culture of NOB has been grown on solid media. NOB streaked onto plates might be inhibited by ambient oxygen (31) or by organic compounds in commonly used solidifying agents (32). Inhibition could also be caused by H_2_O_2_ that is formed when medium containing agar (or agarose) and phosphate is autoclaved (33). No growth of “*Ca*. Nitrotoga” was observed after streaking aliquots of the secondary enrichment onto plaque agarose plates that had been autoclaved in the presence of phosphate and on media containing Noble agar or sieve agarose (with phosphate added before or after autoclaving). In contrast, small (<1 mm), light brown colonies were obtained after incubation for 1 month on plaque agarose medium prepared by the addition of phosphate after autoclaving. Direct Sanger sequencing of 16S rRNA genes PCR amplified from these colonies confirmed that the colonies consisted of “*Ca*. Nitrotoga” cells. The obtained 16S rRNA gene sequence was identical to that retrieved from subsequent liquid cultures (see below and Fig. 1). Thus, selection of a suitable solidifying agent and reduction of H_2_O_2_ formation in the medium were the key prerequisites for growing “*Ca*. Nitrotoga” on plates. A single colony was then restreaked onto new plates, and cells were finally transferred into liquid medium containing nitrite. Subsequent purity checks (see Materials and Methods) confirmed the absence of any other organism in the culture.

Phylogenetic analysis of 16S rRNA genes revealed a close affiliation of the obtained “*Ca*. Nitrotoga” isolate with all other enriched “*Ca*. Nitrotoga” strains and various environmental sequences (Fig. 1). The highest 16S rRNA gene sequence identity shared by the new isolate and a previously enriched “*Ca*. Nitrotoga” member was 98.63% with “*Ca*. Nitrotoga” sp. strain HW29 (25). As this value is below the threshold of 98.7 to 99% used to differentiate species (34) and the obtained isolate showed distinct physiological properties (see below), we propose that this organism represents a separate species within the candidate genus “*Ca*. Nitrotoga.”

**FIG 2:**
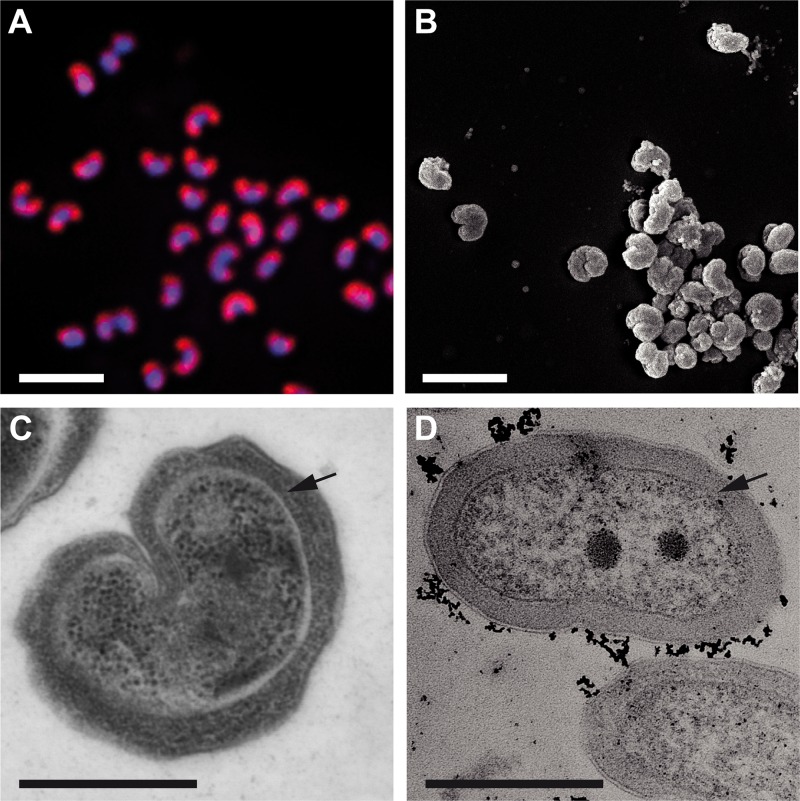
Morphology of “*Ca*. Nitrotoga fabula.” (A) Pure culture of “*Ca*. Nitrotoga fabula” visualized by FISH with the “*Ca*. Nitrotoga”-specific probe Ntoga122 (red) and by DAPI staining (blue). Bar, 2 µm. (B) Scanning electron micrograph imaged after chemical fixation (bar, 2 µm). (C and D) Transmission electron micrographs (after cryopreservation [C] and after chemical fixation [D]; bars, 0.5 µm). Black arrows indicate the periplasmic space.

The new “*Ca*. Nitrotoga” isolate had a peculiar bean-shaped morphology, and the periplasmic space was not enlarged as much as previously described for “*Ca*. Nitrotoga” cells (16, 21, 22) (Fig. 2). Because of the characteristic morphology, we propose the name “*Candidatus* Nitrotoga fabula KNB” (“small bean,” strain designation KNB for the WWTP in Klosterneuburg, Austria) for the new isolate.

### Physiological characterization of “*Ca*. Nitrotoga fabula” in comparison to previous “*Ca*. Nitrotoga” enrichments and other NOB.

**FIG 3:**
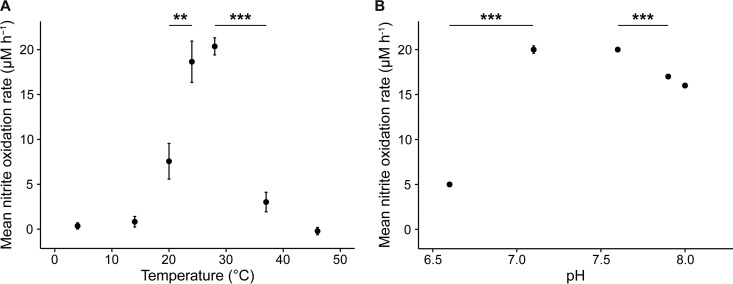
Temperature and pH optima for the nitrite-oxidizing activity of “*Ca*. Nitrotoga fabula.” (A) Mean nitrite oxidation rates during 48 h of incubation at different temperatures. (B) Mean nitrite oxidation rates during 21 h of incubation at different pHs. (A and B) Values are means ± standard deviations (error bars) for three biological replicates. If not visible, error bars are smaller than data points. Values that are significantly different by Welch’s *t* test are indicated by bars and asterisks as follows: **, *P* < 0.01; ***, *P* < 0.001.

The nitrite-oxidizing activity of “*Ca*. Nitrotoga fabula” had its temperature optimum at 24 to 28°C and was poor below 20°C (Fig. 3A). This preference for elevated temperatures was unexpected, because all characterized enriched “*Ca*. Nitrotoga” members prefer lower temperatures or at least remain active under cold conditions (Table 1). The temperature optimum of “*Ca*. Nitrotoga fabula” rather resembles that of some NOB in the genus *Nitrospira* also isolated from WWTPs (32). However, it is noteworthy that uncultured “*Ca*. Nitrotoga” in WWTPs showed activity over a broad range of temperatures from 4 to 27°C (19) (Table 1). Thus, “*Ca*. Nitrotoga” members cover a broad temperature range, and not all species are adapted to low temperature, as was previously assumed for this genus.

**TABLE 1:** Physiological characteristics of isolated or enriched NOB in the candidate genus “*Candidatus* Nitrotoga”^a^

“*Ca*. Nitrotoga” strain	Temp optimum (°C)	pH optimum	Nitrite concn tolerated (mM)	Nitrate concn tolerated (mM)	*K*_*m*(app)_ NO_2_^−^ (µM)
“*Ca*. Nitrotoga fabula” KNB (isolate)	24 to 28 (poor activity at <20)	7.1 to 7.6	Max. concn ND (activity at 1 to 30)	≤25	89.3 ± 3.9
“*Ca*. Nitrotoga arctica” 6680 (enrichment)^b^	10	ND (cultured at 7.4 to 7.6)	<1.2	ND	58 ± 28
“*Ca*. Nitrotoga” sp. strain HAM-1 (enrichment)^c^	ND (cultured at 10 and 17)	ND (cultured at 7.4 to 7.6)	Max. concn ND (cultured at 0.3)	ND	ND
“*Ca*. Nitrotoga” sp. strain AM1 (enrichment)^d^	16	ND (cultured at 8.0 to 8.3)	Max. concn ND (cultured at 0.7 to 2.6)	ND	24.7 ± 9.8
“*Ca*. Nitrotoga” sp. strain HW29 (enrichment)^e^	22 (40% of max. activity at 10)	6.8	<8	ND	ND
Uncultured “*Ca*. Nitrotoga” in WWTPs^f^	Activity at 4 to 27	ND	Activity at 0.1 to 10	ND	ND

a Data for uncultured “*Ca*. Nitrotoga” in activated sludge are listed for comparison. Abbreviations: max., maximum; ND, not determined.

b Data from references 16 and 28.

c Data from reference 21.

d Data from reference 22.

e Data from reference 25.

f Data from reference 19.

The pH optimum of “*Ca*. Nitrotoga fabula” was between 7.1 and 7.6, and activity decreased at more acidic or alkaline conditions (Fig. 3B). Similar to temperature, adaptation to pH varies among “*Ca*. Nitrotoga” members (Table 1). For example, “*Ca*. Nitrotoga” sp. strain HW29 oxidized nitrite most actively at pH 6.8 and retained as much as 75% of its maximal activity at pH 6.1 (25), whereas “*Ca*. Nitrotoga fabula” lost approximately 75% of its maximal activity already at pH 6.6 (Fig. 3B). “*Ca*. Nitrotoga fabula” showed no lag phase of its nitrite-oxidizing activity even with 30 mM nitrite in the medium (see Fig. S1A in the supplemental material), and thus, it tolerated much higher nitrite concentrations than other, enriched “*Ca*. Nitrotoga” members (Table 1). A high tolerance to nitrite was also reported for Nitrospira defluvii (maximum 25 mM) (35) and Nitrolancea hollandica (75 mM), two other NOB isolated from activated sludge (14, 32). Little is known about the nitrate tolerance of “*Ca*. Nitrotoga.” Nitrite oxidation by “*Ca*. Nitrotoga fabula” remained completely inhibited in the presence of >25 mM nitrate even after 1 year of incubation (Fig. S1B).

10.1128/mBio.01186-18.2 FIG S1  Tolerance of “*Ca*. Nitrotoga fabula” to nitrite and nitrate. (A) Mean nitrite oxidation rates with different starting concentrations of nitrite in the medium. The rates were calculated for 7.2 days of incubation. (B) Mean nitrite oxidation rates with different starting concentrations of nitrate in the medium. The rates were calculated between the start of the experiment and depletion of nitrite for setups initially containing 0 or 15 mM nitrate (2.1 and 10.9 days, respectively) and for 13 days of incubation for all other setups. In experiments with starting nitrate concentrations of >25 mM, nitrite was not depleted after 1 year of incubation. (A and B) Data points show the means, and error bars show the standard deviations for three biological replicates (only a single replicate in panel A for 30 mM nitrite). If not visible, error bars are smaller than data points. Download FIG S1, TIF file, 0.4 MB. Copyright © 2018 Kitzinger et al. 2018 Kitzinger et al. This content is distributed under the terms of the Creative Commons Attribution 4.0 International license.

**FIG 4:**
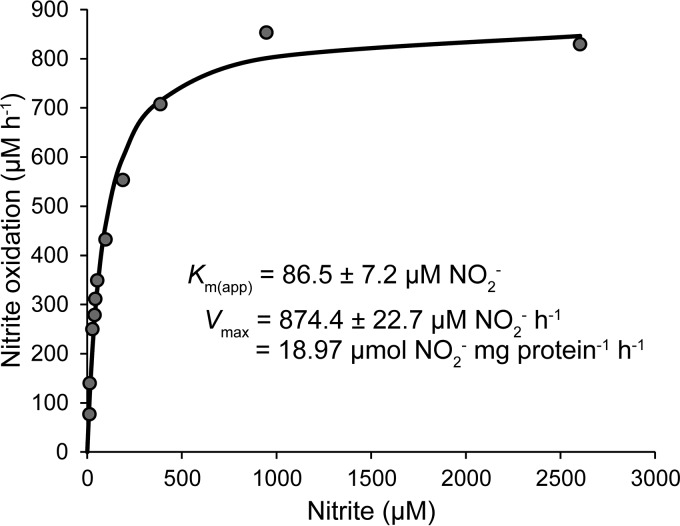
Nitrite oxidation kinetics of “*Ca*. Nitrotoga fabula.” Nitrite oxidation rates were calculated from microsensor measurements of nitrite-dependent O_2_ consumption. The curve indicates the best fit of the data to the Michaelis-Menten kinetic equation. The protein concentration used to calculate *V*_max_ was 46.1 mg/liter. The experiment was performed with biomass concentrated by centrifugation. Data from three additional biological replicates are shown in Fig. S2 in the supplemental material.

Nitrite oxidation by “*Ca*. Nitrotoga fabula” followed Michaelis-Menten kinetics (Fig. 4 and Fig. S2), with a mean apparent half-saturation constant of *K*_*m*(app)_ = 89.3 ± 3.9 µM (standard deviation [SD]) nitrite. The calculated mean maximum oxidation rate of nitrite (*V*_max_) was 27.6 ± 8.4 µmol of nitrite (mg of protein ⋅ h)^−1^ (Fig. 4 and Fig. S2). The measured *K*_*m*(app)_(NO_2_^−^) of “*Ca*. Nitrotoga fabula” was higher (but still in the same order of magnitude) than values reported for “*Ca*. Nitrotoga” enrichments from soil and sediment (Table 1). The slightly poorer affinity for nitrite of “*Ca*. Nitrotoga fabula” may reflect adaptation to different habitats. However, in enrichment cultures, the accompanying organisms may also respire oxygen or use nitrite (e.g., for denitrification) and thus affect affinity measurements based on respirometry (28) or nitrite consumption (22). In either case, the affinity of the NOB can be overestimated in enrichment cultures. Thus, comparison of results obtained by analyses of an isolate and of enrichment cultures must be interpreted with caution. In comparison to other NOB, the affinity for nitrite of “*Ca*. Nitrotoga” is moderate (see Table S1 in the supplemental material). In particular, *Nitrospira* with a significantly higher affinity (Table S1) may outcompete “*Ca*. Nitrotoga” in oligotrophic habitats and in continuously operated WWTPs (which resemble chemostats) where ambient nitrite concentrations are low.

10.1128/mBio.01186-18.3 FIG S2  Nitrite oxidation kinetics of “*Ca*. Nitrotoga fabula.” (A to C) Nitrite oxidation rates were calculated from microsensor measurements of nitrite-dependent O_2_ consumption. The curves indicate the best fit of the data to the Michaelis-Menten kinetic equation. The protein concentrations used to calculate *V*_max_ were 16.17 mg/liter (A), 20.82 mg/liter (B), and 5.17 mg/liter (C). Experiments were performed with concentrated (A and B) or unconcentrated biomass (C). Results for three biological replicates are shown here; a fourth biological replicate is shown in Fig. 4 in the main text. Download FIG S2, TIF file, 0.7 MB. Copyright © 2018 Kitzinger et al. 2018 Kitzinger et al. This content is distributed under the terms of the Creative Commons Attribution 4.0 International license.

10.1128/mBio.01186-18.6 TABLE S1  Kinetic constants of nitrite oxidation of NOB isolates and “*Ca*. Nitrotoga” enrichments. Download TABLE S1, PDF file, 0.1 MB. Copyright © 2018 Kitzinger et al. 2018 Kitzinger et al. This content is distributed under the terms of the Creative Commons Attribution 4.0 International license.

Altogether, adaptations of NOB in the genus “*Ca*. Nitrotoga” to a broad range of conditions (Table 1) likely reflect the wide distribution of this genus in natural and engineered ecosystems.

### Genomic characterization of “*Ca*. Nitrotoga fabula.”

**FIG 5:**
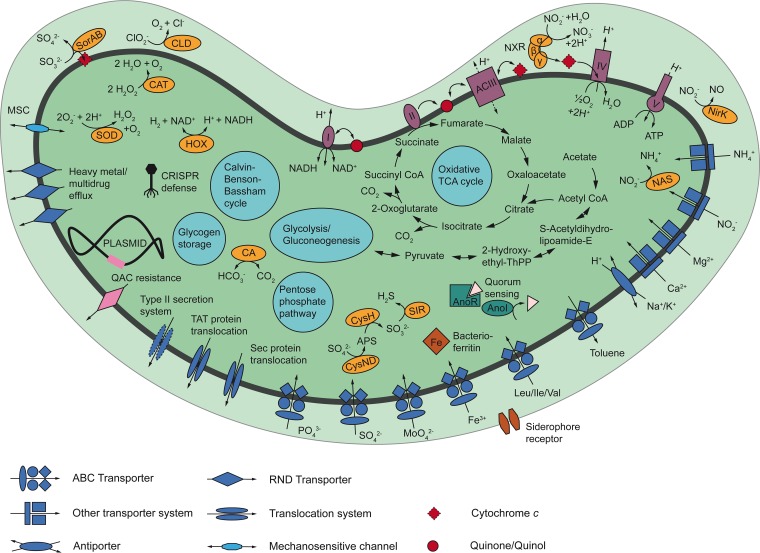
Cell metabolic cartoon constructed from the annotation of the “*Ca*. Nitrotoga fabula” genome. Enzyme complexes of the electron transport chain are labeled with Roman numerals. Table S4 contains further information on the depicted enzymes and pathways. Abbreviations: ACIII, alternative complex III; AnoI/R, acyl-homoserine-lactone synthase/response regulator; CA, carbonic anhydrase; CAT, catalase; CLD, chlorite dismutase; CoA, coenzyme A; CRISPR, clustered regularly interspaced short palindromic repeats; CysH, adenylyl-sulfate reductase; CysND, sulfate adenylyltransferase; HOX, bidirectional group 3d [NiFe] hydrogenase; 2-Hydroxyethyl-ThPP, 2-hydroxyethyl thiamine diphosphate MSC, mechanosensitive channel; NAS, assimilatory nitrite reductase; NirK, nitrite reductase; NXR, nitrite oxidoreductase; RND transporter, resistance-nodulation-cell division transporter; QAC, quaternary ammonium compound; SOD, superoxide dismutase; SIR, assimilatory sulfite reductase; Sor, sulfite:cytochrome *c* oxidoreductase; TAT, twin-arginine translocation; TCA cycle, tricarboxylic acid cycle; Sec, secretion.

The genome of the “*Ca*. Nitrotoga fabula” isolate was completely reconstructed and closed by Illumina and nanopore sequencing (Table S2 and Fig. S3). The chromosome comprises 2,609,426 bp, has an average G+C content of 50.14%, and contains 2,609 coding sequences (CDS). Core metabolic pathways of “*Ca*. Nitrotoga fabula” were reconstructed from the genomic data (Fig. 5 and Table S4). Interestingly, “*Ca*. Nitrotoga fabula” possesses a plasmid that has a size of 5,404 bp and contains six CDS (Tables S2 and S3). Its average G+C content of 63.55% differs drastically from the G+C content of the chromosome, indicating horizontal acquisition of the plasmid. The high similarity of all six CDS to homologs in *Alpha*-, *Beta*-, and *Gammaproteobacteria* (Table S3) suggests a proteobacterial plasmid donor. Plasmids are a rare feature in NOB reported so far only for *Nitrobacter* (36, 37). The plasmid of “*Ca*. Nitrotoga fabula” encodes two hypothetical proteins, a putative transcriptional regulator, a quaternary ammonium compound resistance protein (EmrE), a putative relaxase, and a putative replication initiation protein (Table S3). The latter two are likely involved in plasmid acquisition and replication, respectively. EmrE might be beneficial for life in activated sludge (see below). The plasmid and the capability of “*Ca*. Nitrotoga fabula” to grow on solid media could facilitate the development of a vector and a transformant selection system for using “*Ca*. Nitrotoga fabula” as a genetically tractable model nitrite oxidizer. To date, no genetic system has been established for any NOB.

10.1128/mBio.01186-18.4 FIG S3  Circular representation of the “*Ca*. Nitrotoga fabula” chromosome. Predicted coding sequences (CDS) (rings 1 plus 2), genes of enzymes involved in nitrite oxidation, hydrogen oxidation, and denitrification (ring 3), RNA genes (ring 4), and local nucleotide composition measures (rings 5 plus 6) are shown. Very short features were enlarged to enhance visibility. Clustered genes, such as several tRNA genes, may appear as one line owing to space limitations. The tick interval is 0.2 Mbp. Genes at loci coding for NXR and hydrogenase, and the predicted functions of the respective gene products, are also shown. NirK, Cu-dependent nitrite reductase. Download FIG S3, TIF file, 0.6 MB. Copyright © 2018 Kitzinger et al. 2018 Kitzinger et al. This content is distributed under the terms of the Creative Commons Attribution 4.0 International license.

10.1128/mBio.01186-18.7 TABLE S2  Overview of key features of the “*Ca*. Nitrotoga fabula” KNB genome. Download TABLE S2, PDF file, 0.1 MB. Copyright © 2018 Kitzinger et al. 2018 Kitzinger et al. This content is distributed under the terms of the Creative Commons Attribution 4.0 International license.

10.1128/mBio.01186-18.8 TABLE S3  Proteins encoded by the plasmid of “*Ca*. Nitrotoga fabula” KNB and their closest homologs in the TrEMBL, Swiss-Prot, and NCBI nr databases. Download TABLE S3, PDF file, 0.1 MB. Copyright © 2018 Kitzinger et al. 2018 Kitzinger et al. This content is distributed under the terms of the Creative Commons Attribution 4.0 International license.

10.1128/mBio.01186-18.9 TABLE S4  Proteins of “*Ca*. Nitrotoga fabula” with predicted functions in key metabolic pathways, and genes of “*Ca*. Nitrotoga fabula” coding for rRNAs or tRNAs. Proteins with an amino acid identity of ≥35% (over at least 80% of the sequence lengths) to characterized proteins in the Swiss-Prot or TrEMBL database were annotated as homologous to proteins with a known function. Proteins with an amino acid identity of ≥25% (over at least 80% of the sequence lengths) to characterized proteins or signatures in the aforementioned databases were annotated as putative homologs of the respective database entries. Download TABLE S4, PDF file, 0.2 MB. Copyright © 2018 Kitzinger et al. 2018 Kitzinger et al. This content is distributed under the terms of the Creative Commons Attribution 4.0 International license.

### Nitrite oxidation and nitrite oxidoreductase phylogeny.

**FIG 6:**
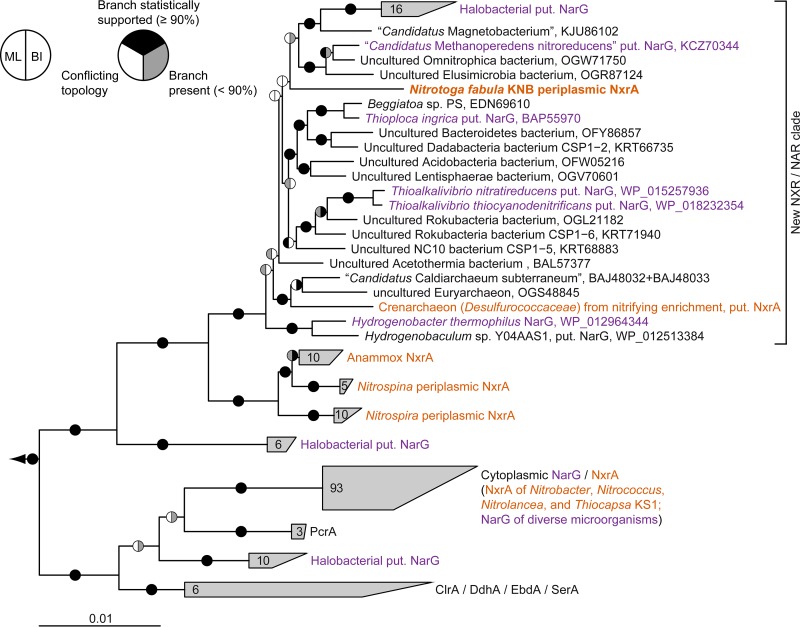
Phylogeny of NxrA from “*Ca*. Nitrotoga fabula” and related proteins. Consensus tree showing the alpha subunits of selected enzymes from the type II DMSO reductase family. Confirmed and putative (put.) NxrA and NarG proteins are indicated. Organisms or enrichment cultures with an observed nitrite-oxidizing phenotype are shown in orange, those with an observed nitrate-reducing phenotype are shown in purple. Pie charts indicate statistical support based on maximum likelihood (ML) (1,000 bootstrap iterations) and Bayesian inference (BI) (posterior probability, 10 independent chains). Numbers in wedges indicate the numbers of taxa. Abbreviations: ClrA, chlorate reductase; DdhA, dimethylsulfide dehydrogenase; EbdA, ethylbenzene dehydrogenase; PcrA, perchlorate reductase; SerA, selenate reductase. Bar, 0.01 estimated substitutions per residue.

Nitrite oxidoreductase (NXR), the key enzyme for nitrite oxidation, belongs to the type II dimethyl sulfoxide (DMSO) reductase family of molybdopterin cofactor-binding enzymes (38, 39). The catalytic alpha subunit (NxrA) of known NXRs contains the Mo cofactor and one Fe-S cluster. It is associated with the beta subunit NxrB, which contains four Fe-S clusters. NxrB likely transfers electrons derived from nitrite to the gamma subunit NxrC or directly to the respiratory chain (39). NXR was reported to be a membrane-associated enzyme (39–41). The proposed membrane anchor is NxrC, which probably binds one or two heme groups and may thus also be involved in electron transfer (39). The three known types of NXR differ in their cellular localization and phylogenetic affiliation (39). In two groups, NxrA and NxrB face the cytoplasmic side of the cell membrane (8, 41). These NXRs are closely related to membrane-bound, cytoplasmically oriented nitrate reductases (NARs) (Fig. 6). One type is found in *Nitrobacter*, *Nitrococcus*, and *Nitrolancea*, and the second type is found in the phototrophic NOB *Thiocapsa* strain KS1 (5, 8, 14, 38) (Fig. 6). In the third group, NxrA and NxrB are oriented toward the periplasmic space. This type occurs in *Nitrospira*, *Nitrospina*, and in anaerobic ammonium oxidizers (anammox organisms) and is phylogenetically distinct from the cytoplasmic NXRs (13, 39, 40) (Fig. 6). In anammox organisms, NXR is localized in the anammoxosome instead of the periplasm (42).

Consistent with its growth on nitrite as the sole energy source and electron donor, “*Ca*. Nitrotoga fabula” encodes NXR (Fig. 5, Fig. S3, and Table S4). The genome contains two identical loci coding for NxrA, NxrB, a putative NxrC, and a putative chaperone (Fig. S3 and Table S4). No other *nxr* genes were identified. NxrA and NxrB of “*Ca*. Nitrotoga fabula” contain the conserved binding sites for the Mo cofactor and the Fe-S clusters found also in the respective homologs of Nitrospira defluvii (39) with only few differences. In NxrA, the Mo binding site has the sequence pattern Y-4x-D-11x-QM instead of Y-4x-D-11x-QN as in N. defluvii. In NxrB of “*Ca*. Nitrotoga fabula,” the binding site of Fe-S cluster IV contains cysteine at the position homologous to Asp45 of N. defluvii and lacks an insertion of eight residues that is found in N. defluvii (39). NxrC shows only low similarity to gamma subunits of other type II DMSO reductase-like enzymes. It contains a predicted heme-binding site but no transmembrane helix, indicating that the NXR of “*Ca*. Nitrotoga fabula” may be soluble or interacts with another membrane-bound protein, as was also discussed for Nitrospina gracilis (13). NxrA contains an N-terminal signal peptide for protein export via the twin-arginine protein translocation mechanism, and NxrC contains an N-terminal signal peptide for translocation via the Sec pathway, suggesting that the NXR of “*Ca*. Nitrotoga fabula” is located in the periplasmic space (Fig. 5). NxrB lacks any translocation signal but may be cotranslocated with NxrA as proposed for the periplasmic NXRs of *Nitrospira* and *Nitrospina* (13, 39). A periplasmic NXR should be energetically advantageous, because nitrite oxidation outside the cell releases protons into the periplasm and may contribute directly to proton motive force (PMF) (39) (Fig. 5). This feature likely helps “*Ca*. Nitrotoga” compete with cooccurring NOB harboring a cytoplasmic NXR.

Intriguingly, phylogenetic analysis of the catalytic NxrA subunit revealed that the NXR of “*Ca*. Nitrotoga fabula” is not closely related to the other known NXR forms. Instead, it belongs to a distinct “sister clade” of the lineage containing the periplasmic NXRs of *Nitrospira*, *Nitrospina*, and anammox (Fig. 6). Some of the proteins affiliated with NxrA of “*Ca*. Nitrotoga fabula” are catalytic subunits of putative NARs (NarG) from phylogenetically diverse bacteria and archaea, which are known nitrate reducers (Fig. 6). Only recently, the enzyme of Hydrogenobacter thermophilus has been functionally characterized as a periplasmically oriented, membrane-bound NAR (43). The affiliation of NXR from “*Ca*. Nitrotoga fabula” with this clade demonstrates that enzymes in this group are capable of nitrite oxidation. Since other NXRs are bidirectional enzymes that oxidize nitrite and also reduce nitrate (4, 5, 44), it is conceivable that known nitrate-reducing members of this clade could also oxidize nitrite for detoxification or even for energy conservation under permissive conditions. An additional requirement for nitrite oxidation would be suitable electron carriers, such as high-potential cytochrome *c* (cyt. *c*), which accept the electrons derived by NXR from nitrite. To our knowledge, except for “*Ca*. Nitrotoga fabula,” none of the cultured organisms possessing enzymes in this NXR/NAR clade has systematically been tested for a nitrite-oxidizing phenotype. Moreover, the clade contains proteins from highly diverse, uncultured, and physiologically uncharacterized organisms (Fig. 6) that might be novel nitrite oxidizers if they also possess high-potential electron carriers. According to this assumption, nitrite oxidation might occur within the domain *Archaea* (Fig. 6). Recently, we sequenced a joint metagenome from pooled DNA from early stage nitrifying enrichments, which had been established at 75°C from a hot spring in Iceland (Text S1). Nitrite oxidation had been observed in several of these cultures. The only *nxr*-like genes found in the assembly were binned into a metagenome-assembled genome of a crenarcheon, which was remotely related to the genus *Ignisphaera* (family *Desulfurococcaceae*) (Fig. S4). Intriguingly, its putative NxrA fell into the same clade as the NxrA of “*Ca*. Nitrotoga fabula.” It grouped with the NxrA/NarG of “*Candidatus* Caldiarchaeum subterraneum” (phylum “Aigarchaeota”), an uncultured and phenotypically uncharacterized thermophilic archaeon (Fig. 6). The absence of unambiguously detected, canonical NOB from the metagenomic data set and the presence of archaea possessing a putative NXR is highly conspicuous and deserves further investigation.

10.1128/mBio.01186-18.1 TEXT S1  In-depth descriptions of protocols used for electron microscopy, for determining the kinetic constants of nitrite oxidation, for sequencing the genome of “*Ca*. Nitrotoga fabula,” and for the establishment and metagenomic analysis of a thermophilic nitrifying enrichment culture. Download TEXT S1, PDF file, 0.1 MB. Copyright © 2018 Kitzinger et al. 2018 Kitzinger et al. This content is distributed under the terms of the Creative Commons Attribution 4.0 International license.

10.1128/mBio.01186-18.5 FIG S4  Metagenomic contig from a crenarcheal sequence bin, which represents a member of the family *Desulfurococcaceae* remotely related to the genus *Ignisphaera*. The contig was retrieved from a metagenomic data set, which had been produced from pooled genomic DNA from thermophilic (75°C) nitrifying enrichments (Text S1). No other sequence bin in the metagenomic data set contained *nxr*-like genes. The predicted gene product of *nxrA* is affiliated with the same phylogenetic clade as the NxrA of “*Ca*. Nitrotoga fabula” in the type II DMSO reductase family (see Fig. 6 in the main text). The putative *nxr* genes are shown in orange, and the flanking archaeal genes are shown in blue. Wiggly lines indicate the ends of the contig. Best BLASTP hits (NCBI nr) for each gene are indicated in brackets together with sequence identity (id), alignment coverage of the query (=contig) sequence (qc), and alignment coverage of the subject (=database) sequence (sc) as a percentage. Genes and noncoding regions are drawn to scale. Download FIG S4, TIF file, 0.6 MB. Copyright © 2018 Kitzinger et al. 2018 Kitzinger et al. This content is distributed under the terms of the Creative Commons Attribution 4.0 International license.

Previous analyses suggested that NXR independently evolved at least three times within the type II DMSO reductase family, leading to the aforementioned three types of cytoplasmic and periplasmic NXRs (8, 39). The distinct phylogenetic position of the novel NXR of “*Ca*. Nitrotoga fabula” indicates an even more complex evolutionary history of nitrite oxidation. Functional data for the enzymes in this clade are too sparse to assess whether nitrite oxidation may be an ancestral feature of this lineage, or more likely a secondary adaptation found in “*Ca*. Nitrotoga” (and possibly additional organisms) with the remaining proteins being strict NARs. However, it is remarkable that the clade shares a common ancestor with the *Nitrospira*/*Nitrospina*/anammox enzymes, which are exclusively NXRs (Fig. 6). It also remains unclear whether this type of NXR evolved in “*Ca*. Nitrotoga” or was acquired through horizontal gene transfer by an ancestor of this genus.

### Central energy and carbon metabolism.

In NOB, electrons derived from nitrite are transferred from NXR to cyt. *c* and then to the terminal oxidase (cyt. *c* oxidase; complex IV) for aerobic respiration (Fig. 5). The genome of “*Ca*. Nitrotoga fabula” encodes several *c*-type cytochromes and a predicted high-affinity, proton-pumping heme-copper cyt. *c* oxidase of the *cbb*_3_ type (Table S4). The conserved energy is used for ATP synthesis by a canonical F_1_F_o_ ATPase (complex V) (Fig. 5 and Table S4). In addition, “*Ca*. Nitrotoga fabula” possesses a canonical NADH dehydrogenase (complex I) and the complete oxidative tricarboxylic acid (TCA) cycle, including a four-subunit succinate dehydrogenase complex (complex II) (Fig. 5 and Table S4). A canonical quinol:cytochrome *c* oxidoreductase (complex III) is lacking, but “*Ca*. Nitrotoga fabula” carries genes encoding an alternative complex III (ACIII) (45) that is highly similar to ACIII of other *Gallionellaceae* members (46). Thus, “*Ca*. Nitrotoga fabula” possesses a complete electron transport chain for respiration using inorganic low-potential electron donors such as H_2_ (see below) or organic compounds. For example, glycogen deposits may serve as an energy source for cell maintenance during starvation (Fig. 5 and Table S4). However, “*Ca*. Nitrotoga fabula” appears to lack genes encoding components in the uptake and utilization of formate, pyruvate, and acetate, which can be used as carbon and/or energy sources by several other NOB (4, 18, 47, 48). A transporter for branched amino acids may enable their use as organic sources of energy, carbon and nitrogen, or directly as protein building blocks (Fig. 5). “*Ca*. Nitrotoga fabula” can also assimilate nitrogen from ammonium and nitrite (Table S4), but in contrast to some other NOB (4, 6), it lacks any known genes for utilizing urea or cyanate.

When nitrite is the sole electron donor, reductants for autotrophic CO_2_ fixation must be provided by reverse electron transport. Unlike *Nitrospira*, “*Ca*. Nitrotoga fabula” lacks multiple copies of complexes I and III that might channel electrons in opposite directions (4, 39). Thus, we assume that these single complexes of “*Ca*. Nitrotoga fabula” are bidirectional and consume PMF for reverse electron transport (Fig. 5). “*Ca*. Nitrotoga fabula” carries genes encoding all the components of the complete Calvin-Benson-Bassham (CBB) cycle for CO_2_ fixation, including two divergent copies of the small (37% amino acid identity) and large (56% identity) subunits of type I ribulose-1,5-bisphosphate carboxylase/oxygenase (RuBisCO). Other NOB using the CBB cycle are *Nitrobacter*, *Nitrococcus*, and *Nitrolancea* (5, 14, 49). In contrast, *Nitrospira* and *Nitrospina* utilize the more oxygen-sensitive reductive TCA cycle (13, 39). Hence, “*Ca*. Nitrotoga” might be more resistant to high dissolved oxygen (DO) concentrations and could have a competitive advantage over *Nitrospira* in strongly aerated activated sludge tanks. Based on its predicted high-affinity terminal oxidase (see above), “*Ca*. Nitrotoga fabula” could also cope with low-DO conditions that occur, for example, in simultaneously nitrifying and denitrifying bioreactors. This may explain the observed presence of “*Ca*. Nitrotoga” in a low-DO nitrifying bioreactor where it cooccurred with *Nitrospira* (50), which can also oxidize nitrite at low DO concentrations (51, 52).

### Alternative energy metabolisms.

The recent discovery that some *Nitrospira* grow chemolithoautotrophically by aerobic hydrogen oxidation was unexpected, because nitrifiers had been regarded as metabolically restricted organisms whose energy metabolism is intimately linked to the nitrogen cycle (3). Interestingly, “*Ca*. Nitrotoga fabula” harbors a complete set of genes encoding a group 3d NAD-coupled [NiFe] hydrogenase and accessory proteins (Fig. S3 and Table S4) (53). The enzymes in this group are cytosolic bidirectional hydrogenases and can be oxygen tolerant (54). The hydrogenase could enable “*Ca*. Nitrotoga fabula” to use H_2_ as an energy source and electron donor for aerobic growth and, if NXR works reversibly, for anaerobic respiration with nitrate as electron acceptor. Both activities were observed for Nitrospira moscoviensis, although growth occurred only in oxic incubations (3, 12). Hydrogenases occur in various NOB (3), and hydrogen oxidation as an alternative energy metabolism has several advantages for these organisms. First, it can help NOB survive nitrite-depleted conditions. Second, electrons derived from H_2_ can be used for CO_2_ fixation without reverse electron transport, saving energy for other cellular functions. Finally, it may enable NOB to colonize niches independent of nitrification. A source of H_2_ could be fermenting heterotrophs living nearby in anoxic niches in soils, sediments, biofilms, and flocs (55).

“*Ca*. Nitrotoga fabula” also carries genes encoding a periplasmic sulfite:cyt. *c* oxidoreductase (Fig. 5 and Table S4), which may allow it to use sulfite as an energy source and electron donor. Recently, the participation of NOB in sulfur cycling was demonstrated for *Nitrococcus* that oxidized sulfide in the presence of O_2_ (5).

### Stress response, defense, and cell-cell communication.

Contrasting their aerobic metabolism, several NOB and also comammox organisms lack catalase, superoxide dismutase, or both (9, 13, 39). “*Ca*. Nitrotoga fabula” possesses both enzymes (Fig. 5 and Table S4) but apparently was nevertheless inhibited by the amount of H_2_O_2_ formed during the preparation of solid media with phosphate (see above).

Wastewater contains many potentially toxic compounds. Accordingly, the genome of “*Ca*. Nitrotoga fabula” encodes various resistance and detoxification mechanisms, including efflux systems for heavy metals and organic solvents, arsenate reductase, and chlorite dismutase (Fig. 5 and Table S4). Quaternary ammonium compounds (QAC) are widely used as disinfectants and are ingredients in cosmetics and household products. In addition to the *emrE* gene on the plasmid (see above), “*Ca*. Nitrotoga fabula” has another QAC resistance gene (*sugE*) on the chromosome. QAC resistance is not a common feature of NOB isolated from WWTPs. While the genome of Nitrospira defluvii encodes SugE, both Nitrospira japonica NJ1 and *Nitrospira* strain ND1 (56) lack QAC resistance genes. The sensitivity of NOB to QAC and other harmful compounds has hardly been studied, but it could be an important factor determining the distribution and abundance of different NOB in sewage treatment systems.

“*Ca*. Nitrotoga fabula” possesses a LuxI/LuxR-type quorum-sensing (QS) system that is similar to the AnoI/AnoR system of Acinetobacter nosocomialis (57) (56% amino acid sequence identity to AnoI and 46% identity to AnoR). QS systems have also been identified in *Nitrobacter* and *Nitrospira* (56, 58). In *Nitrobacter*, QS has been linked to the production and consumption of nitrogen oxides (59). Further functions of QS in NOB await investigation, and it will be exciting to see whether QS plays similar or different roles in phylogenetically diverse NOB, including “*Ca*. Nitrotoga.”

### Description of “*Candidatus* Nitrotoga fabula”.

Fabula (L. fem. noun, small bean, referring to the characteristic bean-shaped morphology of the cells).

Cells are Gram-negative short curved rods with a length of approximately 1 µm and width of approximately 0.5 µm. “*Ca*. Nitrotoga fabula” grows planktonically but forms loose flocs at high cell density. Nonmotile. Aerobic chemolithoautotrophic nitrite oxidizer that uses CO_2_ as the sole carbon source. Temperature optimum of 24 to 28°C, pH optimum of 7.1 to 7.6. Nitrite oxidation was observed up to 30 mM nitrite (higher concentrations not tested) and below 30 mM nitrate. Grows in mineral liquid and on solid (plaque agarose autoclaved without phosphate) media containing nitrite. The genome consists of a single chromosome and a plasmid. The G+C content of the DNA is 50.14 mol% (chromosome) and 63.55 mol% (plasmid).

The “*Ca*. Nitrotoga fabula” KNB strain was isolated from activated sludge of the municipal wastewater treatment plant in Klosterneuburg, Austria. The strain is available from the authors upon request.

### Conclusions.

The physiological and genomic characterization of the first “*Ca*. Nitrotoga” isolate has revealed potential alternative energy metabolisms and a broader spectrum of physiological adaptations in this genus than previously assumed. Like *Nitrospira*, “*Ca*. Nitrotoga” members can be versatile NOB whose metabolic flexibility may explain their competitive success in dynamic environments such as WWTPs. However, fundamental differences between “*Ca*. Nitrotoga” and *Nitrospira* include the affinity for nitrite (Table S1), as well as the resistance of “*Ca*. Nitrotoga” to higher oxygen levels according to the genetic inventory and growth on plates of “*Ca*. Nitrotoga fabula.” Previous studies showed that multiple factors, including the concentrations of DO and nitrite, temperature, and pH, influence the community composition of NOB (21, 25, 50, 51, 60, 61). Further research is needed to understand which conditions in engineered and natural ecosystems allow the coexistence of “*Ca*. Nitrotoga” with *Nitrospira* or other NOB and which factors lead to their competitive exclusion. Intriguingly, the phylogenetic affiliation of the novel NXR of “*Ca*. Nitrotoga” with enzymes from uncharacterized microorganisms indicates that the diversity of nitrite oxidizers in nature might be much larger than currently anticipated.

## MATERIALS AND METHODS

### Sampling and cultivation conditions.

Activated sludge from the combined nitrification/denitrification tank (intermittently aerated; maximum dissolved oxygen [DO] concentration of 2.5 mg/liter) of the municipal wastewater treatment plant (WWTP) in Klosterneuburg, Austria, was sampled in January 2014. The sludge was diluted 1:3,000 in mineral medium that was prepared as described elsewhere (3) and amended with 3 µg Na_2_SeO_3_⋅5H_2_O and 4 µg Na_2_WO_4_⋅2H_2_O per liter. Diluted sludge (150 ml) was inoculated in 300-ml Erlenmeyer flasks that were loosely closed with aluminum caps, supplied with 1 mM NaNO_2_, and incubated at room temperature in darkness and without agitation. Nitrite consumption was regularly monitored by using nitrite-nitrate test strips (Merckoquant; Merck). Upon depletion of nitrite, the cultures were fed with 1 mM NaNO_2_. Aliquots of the enrichments were subcultured into fresh medium (dilution factor of 1:200) in intervals of 3 to 8 weeks. After the second transfer, the cultures were kept in 100-ml borosilicate bottles filled with 40 ml medium and closed with plastic lids.

Solid mineral media containing nitrite were prepared with 1% (wt/vol) Noble agar (catalog no. 214220; Difco), sieve 3:1 agarose (catalog no. 850091; Biozym), or plaque agarose (catalog no. 840100; Biozym). The pH of the medium was adjusted to pH 7.8 either by adding KH_2_PO_4_ prior to the addition of solidifying agent and autoclaving or by adding sterile filtered KH_2_PO_4_ of pH 8 after autoclaving. Aliquots (5 to 10 µl) of 1:100 diluted culture were streaked onto the solid media and incubated at room temperature in darkness for several weeks. Grown colonies were restreaked onto solid medium, and single colonies were finally inoculated into liquid mineral medium. Culture aliquots were cryopreserved in mineral medium containing 10% (vol/vol) dimethyl sulfoxide (DMSO) (62).

### Assessment of culture purity.

The nitrite-oxidizing bacterial (NOB) community composition in liquid enrichment cultures was monitored by rRNA-targeted fluorescence *in situ* hybridization (FISH) after cell fixation in formalin according to standard protocols (63). The oligonucleotide probes applied were Ntspa662 specific for the genus *Nitrospira* (18), Ntoga122 specific for the candidate genus “Nitrotoga” (19), probes EUB338-I to -III that detect most bacteria (64, 65), and NON338 as a control for nonspecific probe binding (66). The probes were 5′ and 3′ doubly labeled with the fluorochromes Fluos, Cy3, and Cy5 and used in combination with the unlabeled competitor oligonucleotides of Ntspa662 and Ntoga122, respectively (18, 19). FISH was combined with nonspecific fluorescent labeling of all cells by 4′,6′-diamidino-2-phenylindole (DAPI). Fluorescence micrographs were recorded using an epifluorescence microscope (Zeiss Axio Imager M2 with AxioCam 506 Mono). The purity of the “*Ca*. Nitrotoga fabula” isolate was assessed by the following: (i) FISH and DAPI staining as described above; (ii) inoculation of Luria-Bertani medium, which was diluted 1:10 in mineral medium, to test for heterotrophic contaminants; and (iii) PCR screening of the culture using the primers 8F and 1492R that target the bacterial 16S rRNA gene (17, 67). After purification (QIAquick PCR purification kit; Qiagen), the PCR products were Sanger sequenced (Microsynth, Austria) without cloning. The purity of the isolate was also confirmed by Illumina sequencing (see below) and by the absence of cells with a divergent morphology in electron micrographs (for a detailed description of sample preparation for electron microscopy, see Text S1 in the supplemental material).

### Physiological experiments.

Cells from pregrown liquid cultures of “*Ca*. Nitrotoga fabula” were collected by centrifugation (8,200 × *g*, 20 min, 20°C). The supernatant was discarded, and the cells were resuspended in fresh mineral medium without nitrite. This procedure was repeated until no nitrite and nitrate was detected in the supernatant. The cells were finally resuspended in fresh mineral medium and served as an inoculum for physiological experiments. All experiments were carried out in biological triplicates. To quantify the nitrite-oxidizing activity, nitrite and nitrate concentrations were measured photometrically as described elsewhere (68, 69). At each time point, samples (0.5 ml) of the incubated cultures were taken, cells were removed by centrifugation (20,100 × *g*, 10 min, 4°C), and the supernatant was stored at −20°C until chemical measurements were performed. To determine the temperature optimum for activity of “*Ca*. Nitrotoga fabula,” 100-ml borosilicate glass bottles containing 40 ml medium supplemented with 1 mM NaNO_2_ were preincubated at the tested temperatures (4 to 46°C). After inoculation with washed cells (see above), the bottles were incubated at the respective temperatures, and the nitrite and nitrate concentrations were quantified after 48 h as described above. To determine the pH optimum for activity of “*Ca*. Nitrotoga fabula,” mineral medium was supplemented with 5 mM (final concentration) sterile-filtered HEPES (Sigma-Aldrich) after autoclaving. The pH was adjusted to 6.6, 7.1, 7.6, 7.9, and 8.1 by adding 1 N NaOH and remained stable throughout the experiment. Samples for nitrite and nitrate concentration measurements were taken during 3 days of incubation at 28°C.

To determine the nitrite and nitrate tolerance, “*Ca*. Nitrotoga fabula” cells were incubated in media containing 1 to 30 mM nitrite or 15 to 50 mM nitrate, respectively, at the optimal temperature (28°C). The media containing nitrate were also supplemented with 1 mM nitrite as the substrate. Nitrite oxidation was then monitored for up to 6 weeks (nitrite tolerance) and up to 1 year (nitrate tolerance).

The nitrite oxidation kinetics of “*Ca*. Nitrotoga fabula” were inferred from instantaneous oxygen uptake measurements in four independent experiments as previously described (70). Nitrite uptake rates were calculated from the measured oxygen uptake rates, and Michaelis-Menten plots of nitrite uptake rates versus nitrite concentration were obtained by fitting a Michaelis-Menten model to the data. Kinetic constants were estimated by nonlinear least-squares regression. For a detailed description of the approach, see Text S1.

### DNA extraction, genome sequencing, and genome annotation.

Cells were collected from a liquid “*Ca*. Nitrotoga fabula” culture, which had been inoculated from a single colony, by centrifugation (8,200 × *g*, 20 min, 20°C) and frozen at −20°C. Total DNA was extracted according to reference 71 with bead beating for cell disruption at 4 m s^−1^. The genome of “*Ca*. Nitrotoga fabula” was sequenced and closed by applying a combination of Illumina and Nanopore technologies (for details of genome sequencing and assembly, please refer to Text S1). The reconstructed genome of “*Ca*. Nitrotoga fabula” was uploaded to the MicroScope platform (72) for automatic annotation and manual annotation refinement of selected metabolic pathways (39).

### Phylogenetic analyses.

Representative full-length 16S rRNA gene sequences classified as “*Ca*. Nitrotoga” in the SILVA Ref NR 99 database (release 132, 13 December 2017) (73) and the 20 top BLASTN hits (>95% alignment coverage, >98% identity) to the 16S rRNA gene sequence of “*Ca*. Nitrotoga fabula” were used to calculate phylogenetic trees. The 16S rRNA gene sequences of cultured *Gallionella* species, and environmental sequences clustering between “*Ca*. Nitrotoga” and *Gallionella*, were used as outgroups. Sequences were aligned using SINA (74); the length of analyzed sequences was between 1,361 and 1,528 bp. Trees were calculated using the neighbor-joining implementation in ARB (75) (Jukes-Cantor substitution model; 1,000 bootstrap iterations) and maximum likelihood algorithms implemented in PhyML (76) and RAxML (77) (gamma model of rate heterogeneity and generalized time-reversible [GTR] substitution model; 1,000 bootstrap iterations). A consensus tree was reconstructed using ARB, and branching patterns were compared manually between all calculated trees. NxrA/NarG protein sequences were aligned using mafft-linsi v.7.312 (78) and trimmed using Trimal v1.4.rev15 (79) with option -automated1. The resulting alignment consisting of 1,206 columns was used to calculate trees in IQ-TREE v1.6.2 (80) and PhyloBayes v4.1b (81). IQ-TREE calculations included model prediction by ModelFinder (82), which identified the best-fit model to be LG+R8, and support values for bipartitions were calculated using UFboot2 (83). PhyloBayes calculations were carried out with 10 independent chains of 5,000 generations using the category (CAT)-GTR model; 2,000 generations of each chain were discarded as burn-in, and the remainder were subsampled every third tree and pooled for calculation of posterior probabilities.

### Accession number(s).

The genome sequence of “*Ca*. Nitrotoga fabula” has been deposited in the European Nucleotide Archive (ENA) under project PRJEB26077. The metagenome-assembled genome sequence of the *Desulfurococcaceae*-related crenarcheon from the thermophilic enrichment has been deposited at NCBI GenBank under project PRJNA461265, accession QFWU00000000.
