# Supplementary figures and images for: Characterization of the First “Candidatus Nitrotoga” Isolate Reveals Metabolic Versatility and Separate Evolution of Widespread Nitrite-Oxidizing Bacteria
Source: mBio. 2018 Jul 10;9(4):e01186-18. doi: 10.1128/mBio.01186-18 (PMC6050957; doi:10.1128/mBio.01186-18)

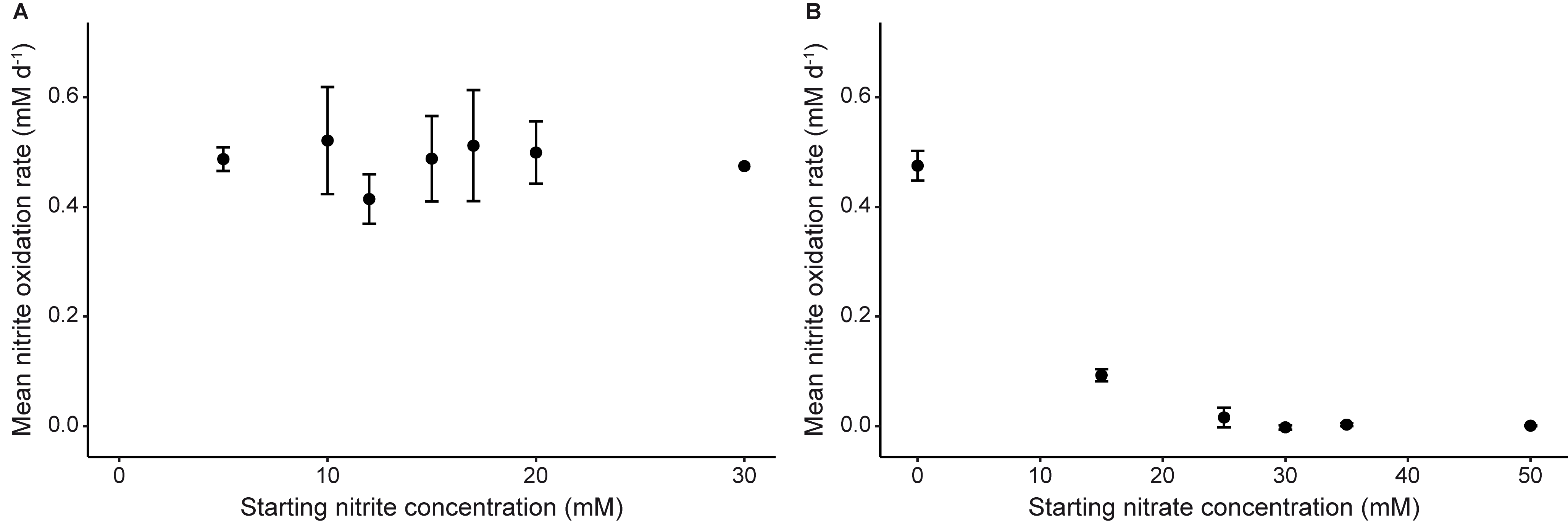

Supplement: FIG S1 [file mbo004183968sf1.tif]

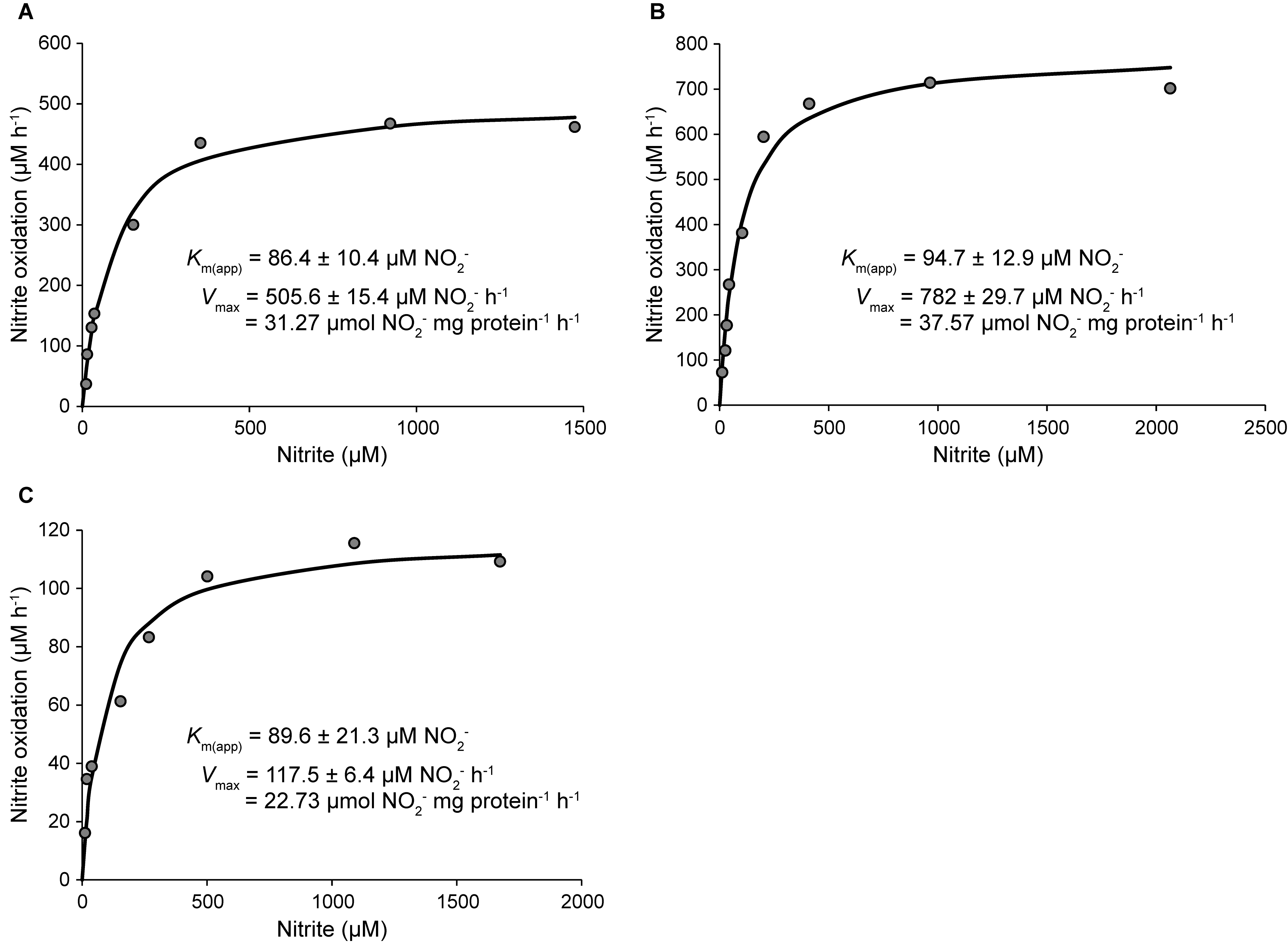

Supplement: FIG S2 [file mbo004183968sf2.tif]

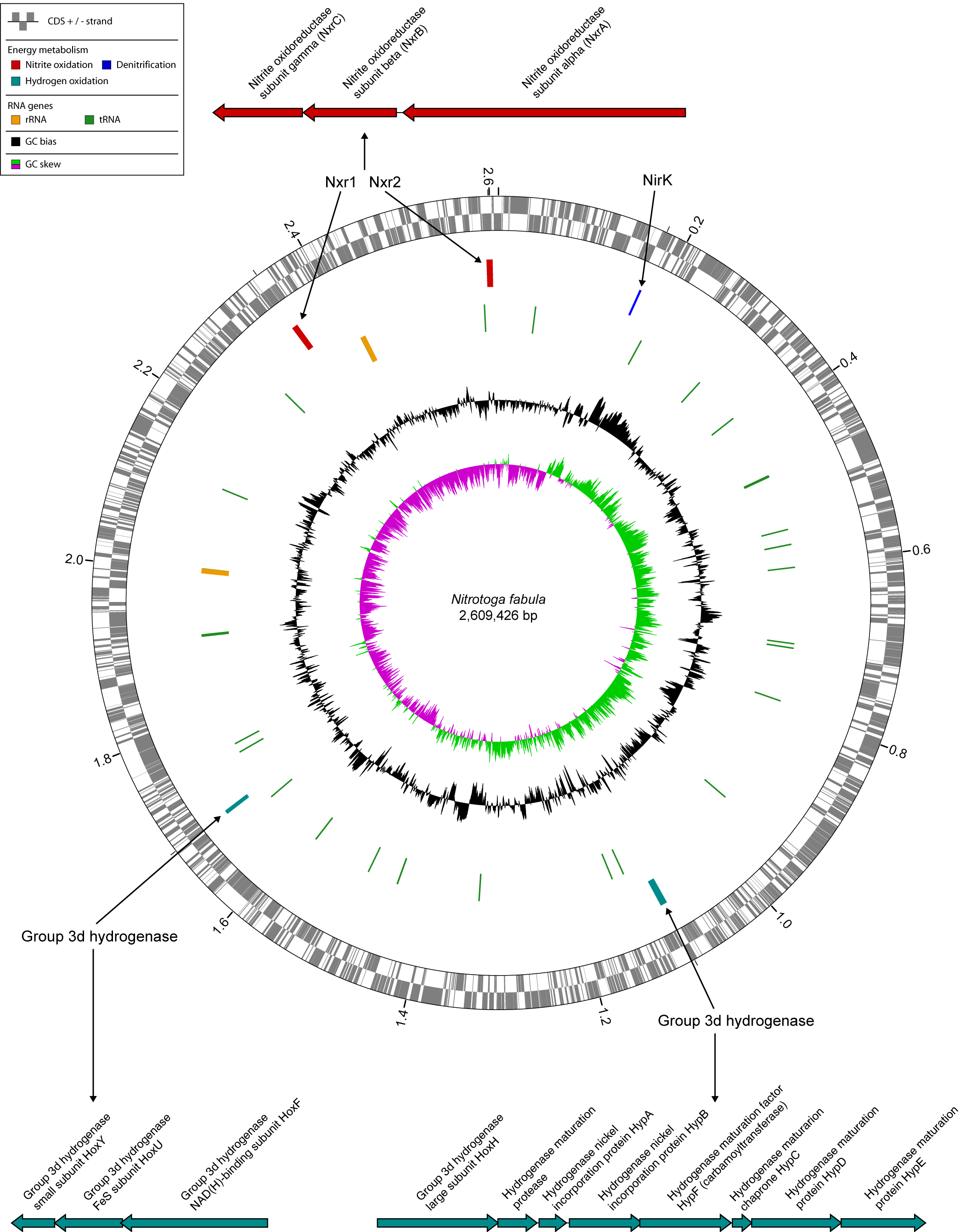

Supplement: FIG S3 [file mbo004183968sf3.tif]

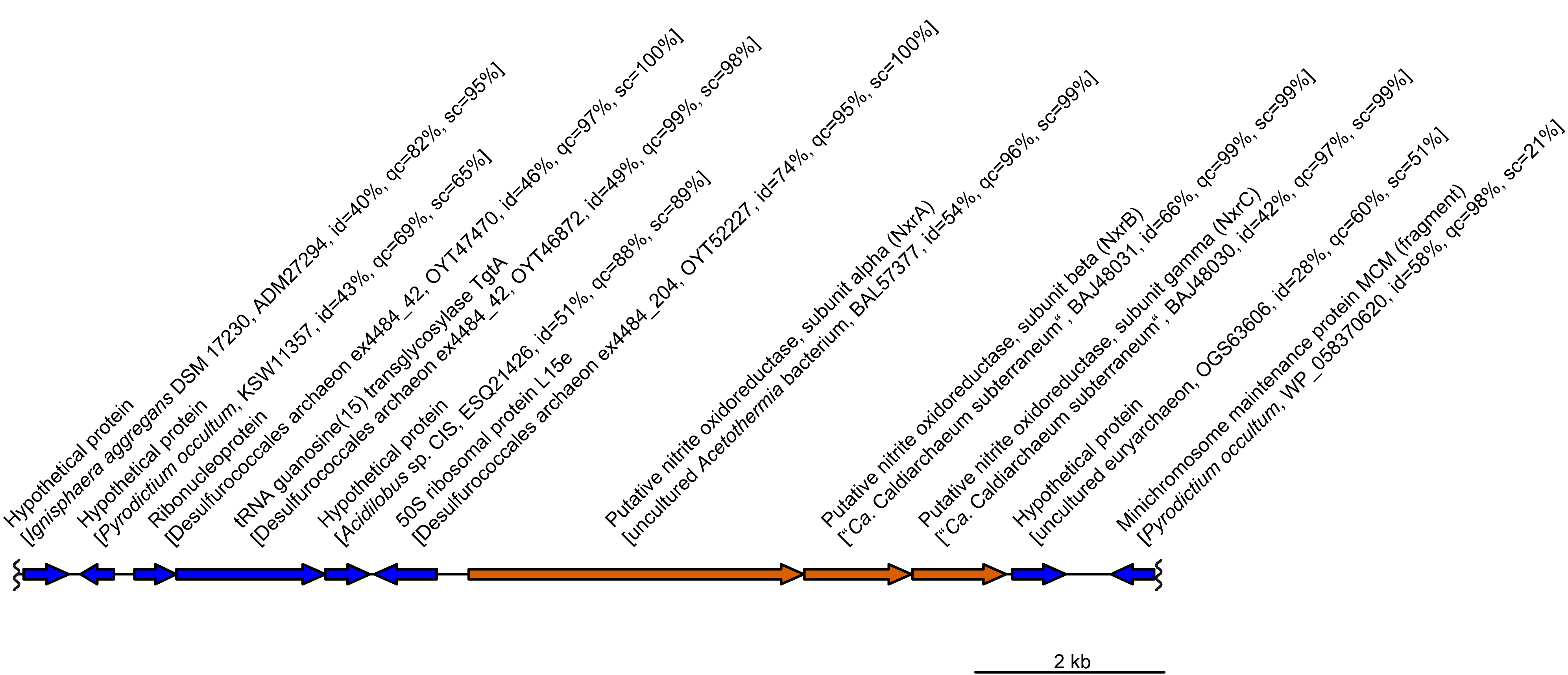

Supplement: FIG S4 [file mbo004183968sf4.tif]
