# Supplementary material for: Characterization of the First “Candidatus Nitrotoga” Isolate Reveals Metabolic Versatility and Separate Evolution of Widespread Nitrite-Oxidizing Bacteria
Source: mBio. 2018 Jul 10;9(4):e01186-18. doi: 10.1128/mBio.01186-18 (PMC6050957; doi:10.1128/mBio.01186-18)
Supplement: TABLE S1 [file mbo004183968st1.pdf]

Table S1. Kinetic constants of nitrite oxidation of NOB isolates and "*Ca*. Nitrotoga" enrichments.

Organism	$K_{m(app)}$ (µM NO_2^-)	V_{max} (µmol NO_2^- mg protein^{-1} h^{-1})	V_{max} (fmol NO_2^- cell^{-1} h^{-1})	Reference
"*Ca.* Nitrotoga fabula KNB" (isolate, WWTP)	89	28	ND[b]	This study
"*Ca*. Nitrotoga arctica" (enrichment, arctic soil)	58	26	ND	(1)
"*Ca*. Nitrotoga sp. AM1" (enrichment, eelgrass sediment)	25	ND	6.1	(2)
Nitrospira defluvii (isolate, WWTP)	9	48	ND	(1)
Nitrospira lenta BS10 (isolate, WWTP)	27	20	ND	(1)
Nitrospira sp. ND1 (isolate, WWTP)	6	45	ND	(3)
Nitrospira japonica NJ1 (isolate, WWTP)	10	31	ND	(3)
Nitrospira moscoviensis (isolate, corroded iron pipe)	9	18	ND	(1)
Nitrospira marina Ecomares 2.1 (isolate, marine aquaculture biofilter)	54	21	ND	(4)
Nitrospira inopinata (isolate, hot groundwater)[a]	449	17	ND	(5)
Nitrobacter hamburgensis (isolate, soil)	540 - 1,370	ND	1 - 3.3	(6)
Nitrobacter hamburgensis (isolate, soil)	544	64	ND	(1)
Nitrobacter winogradskyi (isolate, soil)	36 - 260	ND	1.9 - 3.7	(6)
Nitrobacter winogradskyi (isolate, soil)	309	78	ND	(1)
Nitrobacter vulgaris (isolate, sewage)	49	164	ND	(1)
Nitrobacter sp. 311 (isolate, ocean surface water)	28	95	ND	(4)
Nitrolancea hollandica Lb (isolate, WWTP)	1,000	ND	ND	(7)
Nitrococcus mobilis 231 (isolate, ocean surface water)	120	141	ND	(4)
Nitrospina watsonii 347 (isolate, Black Sea)	19	37	ND	(4)

[a]*N. inopinata* is a complete ammonia oxidizer (comammox organism).

[b]ND=not determined.

References for Table S1

1. Nowka B, Daims H, Spieck E. 2015. Comparison of oxidation kinetics of nitrite-oxidizing bacteria: nitrite availability as a key factor in niche differentiation. Appl Environ Microbiol 81:745-753.

2. Ishii K, Fujitani H, Soh K, Nakagawa T, Takahashi R, Tsuneda S. 2017. Enrichment and physiological characterization of a cold-adapted nitrite-oxidizing *Nitrotoga* sp from an eelgrass sediment. Appl Environ Microbiol 83:e00549-17.

3. Ushiki N, Jinno M, Fujitani H, Suenaga T, Terada A, Tsuneda S. 2017. Nitrite oxidation kinetics of two *Nitrospira* strains: The quest for competition and ecological niche differentiation. J Biosci Bioeng 123:581-589.

4. Jacob J, Nowka B, Merten V, Sanders T, Spieck E, Dahnke K. 2017. Oxidation kinetics and inverse isotope effect of marine nitrite-oxidizing isolates. Aquat Microb Ecol 80:289-300.

5. Kits KD, Sedlacek CJ, Lebedeva EV, Han P, Bulaev A, Pjevac P, Daebeler A, Romano S, Albertsen M, Stein LY, Daims H, Wagner M. 2017. Kinetic analysis of a complete nitrifier reveals an oligotrophic lifestyle. Nature 549:269-272.

6. Both GJ, Gerards S, Laanbroek HJ. 1992. Kinetics of nitrite oxidation in two *Nitrobacter* species grown in nitrite-limited chemostats. Arch Microbiol 157:436-441.

7. Sorokin D, Lücker S, Vejmelkova D, Kleerbezem R, Muyzer G, Sinninghe-Damsté J, Wagner M, Le Paslier D, van Loosdrecht M, Daims H. 2012. Nitrification expanded: Discovery, physiology, and genomics of a nitrite-oxidizing bacterium from the phylum *Chloroflexi*. ISME J 6:2245-2256.
