# Supplementary material for: Characterization of the First “Candidatus Nitrotoga” Isolate Reveals Metabolic Versatility and Separate Evolution of Widespread Nitrite-Oxidizing Bacteria
Source: mBio. 2018 Jul 10;9(4):e01186-18. doi: 10.1128/mBio.01186-18 (PMC6050957; doi:10.1128/mBio.01186-18)
Supplement: TABLE S2 [file mbo004183968st2.pdf]

Table S2. Overview of key features of the "*Ca*. N. fabula KNB" genome.

	Chromosome	Plasmid
Genome size	2,609,426 bp	5,404 bp
Average G+C content	50.14%	63.55%
Number of genomic objects [CDS, fragment CDS, (r,t)RNA]	2,664	6
Number of coding sequences (CDS)	2,609	6
rRNA operons	2	0
tRNA genes	42	0
Coding density	84.98%	62.71%
Repeated regions	5.04%	0

Chromosome clusters of orthologous groups (COG) automated classification

Functional category		CDS	CDS (%)
D	Cell cycle control, cell division, chromosome partitioning	38	1.46
M	Cell wall/membrane/envelope biogenesis	174	6.69
N	Cell motility	14	0.54
O	Posttranslational modification, protein turnover, chaperones	126	4.84
T	Signal transduction mechanisms	65	2.50
U	Intracellular trafficking, secretion, and vesicular transport	60	2.31
V	Defense mechanisms	36	1.38
A	RNA processing and modification	1	0.04
J	Translation, ribosomal structure and biogenesis	151	5.81
K	Transcription	100	3.85
L	Replication, recombination and repair	170	6.54
C	Energy production and conversion	125	4.81
E	Amino acid transport and metabolism	154	5.92
F	Nucleotide transport and metabolism	57	2.19
G	Carbohydrate transport and metabolism	87	3.35
H	Coenzyme transport and metabolism	106	4.08
I	Lipid transport and metabolism	49	1.88
P	Inorganic ion transport and metabolism	130	5.00
Q	Secondary metabolites biosynthesis, transport and catabolism	38	1.46
