# Supplementary material for: Characterization of the First “Candidatus Nitrotoga” Isolate Reveals Metabolic Versatility and Separate Evolution of Widespread Nitrite-Oxidizing Bacteria
Source: mBio. 2018 Jul 10;9(4):e01186-18. doi: 10.1128/mBio.01186-18 (PMC6050957; doi:10.1128/mBio.01186-18)
Supplement: TABLE S3 [file mbo004183968st3.pdf]

Table S3. Proteins encoded by the plasmid of "*Ca*. N. fabula KNB" and their closest homologs in the TrEMBL, Swiss-Prot, and NCBI nr databases.

Plasmid Gene	Predicted function	Database	Best hit, accession no.	Best hit, identity (%)	Best hit, predicted function	Best hit, organism
NITFABP_0001	Protein of unknown function	TrEMBL	A0A0F3GCT4	69	Uncharacterized protein	*Pseudomonas pseudoalcaligenes*
		Swiss-Prot	P20085	49	Mobilization protein MobL	*Acidithiobacillus ferrooxidans*
		nr	XP_012260969	99	uncharacterized protein LOC105688902	*Athalia rosae*[a]
NITFABP_0002	Putative protein involved in initiation of plasmid replication	TrEMBL	A0A1I3FIQ5	83	Initiator Replication protein	*Paracoccus aminovorans*
		Swiss-Prot	P17492	29	Replication protein	*Neisseria gonorrhoeae*
		nr	XP_012260970	100	uncharacterized protein LOC105688903	*Athalia rosae*[a]
NITFABP_0003	Conserved protein of unknown function	TrEMBL	A0A212B9I4	71	Uncharacterized protein	*Pseudomonas* sp. A46
		Swiss-Prot	No hit			
		nr	WP_088193532	71	hypothetical protein	*Pseudomonas* sp. A46
NITFABP_0004	Putative transcriptional regulator, TetR family	TrEMBL	A7KK53	100	Putative transcriptional regulator	*Delftia acidovorans*
		Swiss-Prot	P39897	34	HTH-type transcriptional regulator MtrR	*Neisseria gonorrhoeae*
		nr	WP_043008328	100	TetR/AcrR family transcriptional regulator	*Comamonas testosteronii*
NITFABP_0005	Quaternary ammonium compound-resistance protein EmrE	TrEMBL	A7TX95	100	Small multidrug resistance protein	*Delftia tsuruhatensis*
		Swiss-Prot	Q9X2N9	59	Quaternary ammonium compound-resistance protein QacF	*Klebsiella aerogenes*
		nr	WP_043008326	100	quaternary ammonium compound efflux SMR transporter QacF	*Comamonas testosteronii*
NITFABP_0006	Putative Mobilization protein, MobS-like	TrEMBL	A0A238DWW0	84	Uncharacterized protein	*Thiomonas* sp. X19
		Swiss-Prot	P20086	51	Mobilization protein MobS	*Acidithiobacillus ferrooxidans*
		nr	ART89884	70	hypothetical protein	uncultured bacterium

[a]The presence of these genes in the genome sequence of the insect *A. rosae* is interesting. However, since all genes on the plasmid of "*Ca*. N. fabula" have close homologs in members of the Proteobacteria, we consider the plasmid to be of proteobacterial origin.
