# Supplementary material for: Characterization of the First “Candidatus Nitrotoga” Isolate Reveals Metabolic Versatility and Separate Evolution of Widespread Nitrite-Oxidizing Bacteria
Source: mBio. 2018 Jul 10;9(4):e01186-18. doi: 10.1128/mBio.01186-18 (PMC6050957; doi:10.1128/mBio.01186-18)
Supplement: TABLE S4 [file mbo004183968st4.pdf]

Table S4. Proteins of "*Ca*. N. fabula" with predicted functions in key metabolic pathways, and genes of "*Ca*. N. fabula" coding for rRNAs or tRNAs. Proteins with an amino acid identity >=35% (over at least 80% of the sequence lengths) to characterized proteins in the SwissProt or TrEMBL databases were annotated as homologous to proteins with a known function. Proteins with an amino acid identity >=25% (over at least 80% of the sequence lengths) to characterized proteins or signatures in the aforementioned databases were annotated as putative homologs of the respective database entries.

**Central Carbon Metabolism**						
**Pathway**	**Identifier**	**Type**	**Begin**	**End**	**Gene**	**Product**
C-fixation	NITFAB_0176	CDS	159745	160809	*fda*	Fructose-1,6-bisphosphate aldolase, class II
C-fixation	NITFAB_0177	CDS	160899	162338	*pykA*	Pyruvate kinase
C-fixation	NITFAB_0178	CDS	162355	163539	*pgk*	Phosphoglycerate kinase
C-fixation	NITFAB_0179	CDS	163617	164612	*gapA*	Glyceraldehyde-3-phosphate dehydrogenase A
C-fixation	NITFAB_0181	CDS	165202	167217	*tktA*	Transketolase 1, thiamin-binding
C-fixation	NITFAB_0347	CDS	350588	353368	*ppc*	Phosphoenolpyruvate carboxylase
C-fixation	NITFAB_0411	CDS	418433	419437	*fbp*	Fructose-1,6-bisphosphatase class I
C-fixation	NITFAB_0432	CDS	441142	442029	*cbbP*	Phosphoribulokinase
C-fixation	NITFAB_1072	CDS	1103461	1105800	*cbbO*	von Willebrand factor type A
C-fixation	NITFAB_1073	CDS	1105873	1106688	*cbbQ*	CbbQ
C-fixation	NITFAB_1074	CDS	1106824	1107180	*cbbS1*	Ribulose bisphosphate carboxylase/oxygenase small chain
C-fixation	NITFAB_1075	CDS	1107243	1108664	*cbbL1*	Ribulose bisphosphate carboxylase/oxygenase large chain
C-fixation	NITFAB_1105	CDS	1141097	1141852	*tpiA*	Triosephosphate isomerase
C-fixation	NITFAB_1227	CDS	1242715	1243149	*cbbS2*	Ribulose bisphosphate carboxylase/oxygenase small chain
C-fixation	NITFAB_1228	CDS	1243171	1244646	*cbbL2*	Ribulose bisphosphate carboxylase/oxygenase large chain
C-fixation	NITFAB_1234	CDS	1251630	1254005	*xpkA*	Xylulose-5-phosphate phosphoketolase
C-fixation	NITFAB_1933	CDS	1931517	1932245	*rpe*	Ribulose-phosphate 3-epimerase
C-fixation	NITFAB_2483	CDS	2497131	2497790	*rpiA*	Ribose 5-phosphate isomerase, constitutive
Glycogen formation/degradation	NITFAB_0535	CDS	533427	534872	*glgA*	Glycogen synthase
Glycogen formation/degradation	NITFAB_0599	CDS	592567	593943	*pgm*	Phosphoglucomutase
Glycogen formation/degradation	NITFAB_2047	CDS	2053431	2054711	*glgC*	glucose-1-phosphate adenylyltransferase
Glycogen formation/degradation	NITFAB_2048	CDS	2054819	2057017	*glgB*	1,4-alpha-glucan branching enzyme
Glycogen formation/degradation	NITFAB_2215	CDS	2227727	2229445		Putative Phosphorylase
Glycolysis/Gluconeogenesis	NITFAB_0176	CDS	159745	160809	*fda*	Fructose-1,6-bisphosphate aldolase, class II
Glycolysis/Gluconeogenesis	NITFAB_0177	CDS	160899	162338	*pykA*	Pyruvate kinase
Glycolysis/Gluconeogenesis	NITFAB_0178	CDS	162355	163539	*pgk*	Phosphoglycerate kinase
Glycolysis/Gluconeogenesis	NITFAB_0179	CDS	163617	164612	*gapA*	Glyceraldehyde-3-phosphate dehydrogenase A
Glycolysis/Gluconeogenesis	NITFAB_0411	CDS	418433	419437	*fbp*	Fructose-1,6-bisphosphatase class I
Glycolysis/Gluconeogenesis	NITFAB_0599	CDS	592567	593943	*pgm*	Phosphoglucomutase
Glycolysis/Gluconeogenesis	NITFAB_0652	CDS	652423	653409	*glk*	Glucokinase
Glycolysis/Gluconeogenesis	NITFAB_1105	CDS	1141097	1141852	*tpiA*	Triosephosphate isomerase
Glycolysis/Gluconeogenesis	NITFAB_1993	CDS	1995346	1996629	*eno*	enolase

Glycolysis/Gluconeogenesis	NITFAB_2049	CDS	2057251	2058894	*pgi*	glucosephosphate isomerase
Glycolysis/Gluconeogenesis	NITFAB_2281	CDS	2292994	2294520	*gpmI*	phosphoglycerat mutase, cofactor-independent
Glycolysis/Gluconeogenesis	NITFAB_2363	CDS	2367922	2369181		putative Pyrophosphate--fructose 6-phosphate 1-phosphotransferase
Glyoxylate Shunt	NITFAB_0625	CDS	618890	619543	*gph2*	Phosphoglycolate phosphatase
Glyoxylate Shunt	NITFAB_0676	CDS	687556	689046	*glcD*	Glycolate oxidase GlcD subunit, FAD-linked
Glyoxylate Shunt	NITFAB_0677	CDS	689046	690110	*glcE*	Glycolate oxidase FAD binding GlcE subunit
Glyoxylate Shunt	NITFAB_0678	CDS	690118	691335	*glcF*	glycolate oxidase iron-sulfur GlcF subunit
Glyoxylate Shunt	NITFAB_2021	CDS	2026998	2027972	*mcl*	Malyl-CoA lyase
Glyoxylate Shunt	NITFAB_2022	CDS	2027982	2029160	*mtkA*	Probable Malate--CoA ligase, subunit beta
Glyoxylate Shunt	NITFAB_2023	CDS	2029182	2030054	*sucD*	Probable Malate--CoA ligase, alpha subunit
Glyoxylate Shunt	NITFAB_2118	CDS	2126338	2127354	*mdh*	Malate dehydrogenase
Pentose-phosphate pathway	NITFAB_0176	CDS	159745	160809	*fda*	Fructose-1,6-bisphosphate aldolase, class II
Pentose-phosphate pathway	NITFAB_0181	CDS	165202	167217	*tktA*	Transketolase, thiamin-binding
Pentose-phosphate pathway	NITFAB_0411	CDS	418433	419437	*fbp*	Fructose-1,6-bisphosphatase class I
Pentose-phosphate pathway	NITFAB_0549	CDS	549127	550080	*prs*	ribose-phosphate pyrophosphokinase (Phosphoribosyl pyrophosphate synthetase)
Pentose-phosphate pathway	NITFAB_0599	CDS	592567	593943	*pgm*	Phosphoglucomutase
Pentose-phosphate pathway	NITFAB_0788	CDS	813734	814645		putative 6-phosphogluconate dehydrogenase
Pentose-phosphate pathway	NITFAB_1234	CDS	1251630	1254005	*xpkA*	Xylulose-5-phosphate phosphoketolase
Pentose-phosphate pathway	NITFAB_2044	CDS	2048141	2048860		putative 6-phosphogluconolactonase
Pentose-phosphate pathway	NITFAB_2049	CDS	2057251	2058894	*pgi*	glucosephosphate isomerase
Pentose-phosphate pathway	NITFAB_2453	CDS	2465032	2465793	*rpe*	D-ribulose-5-phosphate 3-epimerase
Pentose-phosphate pathway	NITFAB_2473	CDS	2486672	2487757		putative Transaldolase
Pentose-phosphate pathway	NITFAB_2474	CDS	2487861	2489324	*zwf*	Glucose-6-phosphate 1-dehydrogenase
Pentose-phosphate pathway	NITFAB_2483	CDS	2497131	2497790	*rpiA*	Ribose 5-phosphate isomerase, constitutive
TCA Cycle	NITFAB_0684	CDS	698060	698434	*sdhC*	Succinate dehydrogenase, cytochrome b556 subunit
TCA Cycle	NITFAB_0685	CDS	698428	698802	*sdhD*	Succinate dehydrogenase, hydrophobic membrane anchor protein
TCA Cycle	NITFAB_0686	CDS	698802	700565	*sdhA*	Succinate dehydrogenase, flavoprotein subunit
TCA Cycle	NITFAB_0687	CDS	700925	701620	*sdhB*	Succinate dehydrogenase, iron-sulfur subunit
TCA Cycle	NITFAB_0689	CDS	701857	703200	*gltA*	Citrate synthase
TCA Cycle	NITFAB_0690	CDS	703316	706222	*sucA*	2-oxoglutarate dehydrogenase, E1 component
TCA Cycle	NITFAB_0691	CDS	706240	707400	*sucB*	2-oxoglutarate dehydrogenase, dihydrolipoyllysine-residue succinyltransferase (E2) component
TCA Cycle	NITFAB_0692	CDS	707529	708953	*lpdG*	2-oxo-acid dehydrogenase, dihydrolipoamide dehydrogenase (E3) component
TCA Cycle	NITFAB_1259	CDS	1283730	1284977	*icd*	isocitrate dehydrogenase
TCA Cycle	NITFAB_1443	CDS	1484837	1486219	*fumC*	fumarate hydratase (fumarase C), aerobic Class II
TCA Cycle	NITFAB_2118	CDS	2126338	2127354	*mdh*	Malate dehydrogenase
TCA Cycle	NITFAB_2202	CDS	2216587	2217468	*sucD*	Succinyl-CoA ligase [ADP-forming], subunit alpha
TCA Cycle	NITFAB_2203	CDS	2217456	2218634	*sucC*	Succinyl-CoA ligase [ADP-forming] subunit beta

TCA Cycle	NITFAB_2306	CDS	2313435	2316161	*acnA*	aconitate hydratase 1
TCA Cycle	NITFAB_2386	CDS	2393636	2395429	*lpdA*	2-oxo-acid dehydrogenase complex, Dihydrolipoamide dehydrogenase (E3) component
**Hydrogen Metabolism**						
**Pathway**	**Label**	**Type**	**Begin**	**End**	**Gene**	**Product**
Hydrogen Metabolism	NITFAB_0289	CDS	287290	287868	*hupE*	Hydrogenase/urease accessory protein HupE/UreJ
Hydrogen Metabolism	NITFAB_1062	CDS	1092550	1094052	*hoxH*	[NiFe] NAD-reducing hydrogenase, large subunit HoxH
Hydrogen Metabolism	NITFAB_1063	CDS	1094046	1094534	*hoxW*	Hydrogenase maturation protease
Hydrogen Metabolism	NITFAB_1064	CDS	1094558	1094899	*hypA*	Hydrogenase nickel incorporation protein HypA
Hydrogen Metabolism	NITFAB_1065	CDS	1094936	1095838	*hypB*	Hydrogenase nickel incorporation protein HypB
Hydrogen Metabolism	NITFAB_1066	CDS	1095810	1096946	*hypF*	Carbamoyltransferase HypF (fragment)
Hydrogen Metabolism	NITFAB_1067	CDS	1096960	1097202	*hypC*	Hydrogenase expression/formation protein HypC
Hydrogen Metabolism	NITFAB_1068	CDS	1097199	1098314	*hypD*	Hydrogenase expression/formation protein HypD
Hydrogen Metabolism	NITFAB_1069	CDS	1098311	1099360	*hypE*	Hydrogenase maturation protein HypE
Hydrogen Metabolism	NITFAB_1663	CDS	1683318	1683854	*hoxY*	[NiFe] NAD-reducing hydrogenase, small subunit HoxY
Hydrogen Metabolism	NITFAB_1664	CDS	1683851	1684696	*hoxU*	[NiFe] NAD-reducing hydrogenase, subunit HoxU
Hydrogen Metabolism	NITFAB_1665	CDS	1684672	1686498	*hoxF*	[NiFe] NAD-reducing hydrogenase, subunit HoxF
**Miscellaneous**						
**Pathway**	**Label**	**Type**	**Begin**	**End**	**Gene**	**Product**
Amino Acid Transport	NITFAB_1244	CDS	1265623	1268685		putative Amino acid ABC transporter, permease protein, 3-TM region, His/Glu/Gln/Arg/opine family
Amino Acid Transport	NITFAB_1245	CDS	1268682	1269785		putative ABC-type polar amino acid transport system, ATPase component
Arsenic Resistance	NITFAB_1123	CDS	1150900	1151916		Transcriptional regulator, ArsR family
Arsenic Resistance	NITFAB_1500	CDS	1547297	1547692	*arsC*	arsenate reductase
Arsenic Resistance	NITFAB_1579	CDS	1615184	1615585		Transcriptional regulator, ArsR family
Arsenic Resistance	NITFAB_2149	CDS	2162629	2162961		putative Transcriptional regulator, ArsR family
Arsenic Resistance	NITFAB_2150	CDS	2162958	2163725		putative NADPH-dependent FMN reductase, Arsenical resistance protein
Arsenic Resistance	NITFAB_2151	CDS	2163722	2164228		putative Arsenate reductase
Arsenic Resistance	NITFAB_2217	CDS	2230354	2230785		putative Transcriptional regulator, ArsR family
Branched AA Transport	NITFAB_0006	CDS	5844	7028	*livK*	leucine/isoleucine/valine transporter subunit; periplasmic-binding component of ABC superfamily
Branched AA Transport	NITFAB_0007	CDS	7088	8011	*livH*	leucine/isoleucine/valine transporter subunit; membrane component of ABC superfamily
Branched AA Transport	NITFAB_0008	CDS	7995	9107	*livM*	leucine/isoleucine/valine transporter subunit; membrane component of ABC superfamily
Branched AA Transport	NITFAB_0009	CDS	9113	9922	*livG*	leucine/isoleucine/valine transporter subunit; ATP-binding component of ABC superfamily
Branched AA Transport	NITFAB_0010	CDS	9962	10690	*livF*	leucine/isoleucine/valine transporter subunit; ATP-binding component of ABC superfamily
Calcium Transport	NITFAB_0651	CDS	649611	652247	*tcaB*	Calcium-transporting ATPase

Calcium Transport	NITFAB_1239	CDS	1260412	1261389		putative Sodium/potassium/calcium antiporter CaxA
Carbonic Anhydrases	NITFAB_0956	CDS	995979	996668	*cynT1*	Carbonic anhydrase
Carbonic Anhydrases	NITFAB_1300	CDS	1326582	1327205	*cynT2*	Carbonic anhydrase
Carbonic Anhydrases	NITFAB_1418	CDS	1463115	1463750		putative Carbonic anhydrase
Catalase	NITFAB_1016	CDS	1046232	1048457	*katG*	catalase/hydroperoxidase HPI(I)
Catalase	NITFAB_1541	CDS	1584179	1586377	*katG*	catalase/hydroperoxidase HPI(I)
Catalase	NITFAB_1652	CDS	1667427	1669601	*katG*	catalase/hydroperoxidase HPI(I)
Chlorite Dismutase	NITFAB_1630	CDS	1651185	1652057	*cld*	Chlorite dismutase
Chlorite Dismutase	NITFAB_2070	CDS	2082825	2083667	*cld*	Chlorite dismutase
Co/Zn/Cd Export	NITFAB_1397	CDS	1438159	1441356	*czcA*	Cobalt-zinc-cadmium resistance protein CzcA (Cation efflux system protein CzcA)
Co/Zn/Cd Export	NITFAB_1398	CDS	1441377	1442879	*czcB*	Cobalt-zinc-cadmium resistance protein CzcB
Co/Zn/Cd Export	NITFAB_1399	CDS	1442917	1444209	*czcC*	Cobalt-zinc-cadmium resistance protein CzcC
CRISPR/CAS	NITFAB_1828	CDS	1819684	1820550		putative CRISPR-associated RAMP protein, Cmr4 family
CRISPR/CAS	NITFAB_2256	CDS	2265092	2265397		putative CRISPR-associated endoribonuclease Cas2
CRISPR/CAS	NITFAB_2257	CDS	2265425	2266354		putative CRISPR-associated endonuclease Cas1
CRISPR/CAS	NITFAB_2258	CDS	2266360	2268777		putative CRISPR-associated endonuclease, cas9
Cytochrome c biogenesis	NITFAB_0081	CDS	70926	72869	*ccmF*	heme lyase, CcmF subunit
Cytochrome c biogenesis	NITFAB_0082	CDS	72881	73408	*ccmG*	periplasmic thioredoxin of cytochrome c-type biogenesis
Cytochrome c biogenesis	NITFAB_0083	CDS	73398	73892	*ccmH*	Cytochrome C biogenesis protein
Cytochrome c biogenesis	NITFAB_0084	CDS	73889	75145		putative Cytochrome c-type biogenesis protein
Cytochrome c biogenesis	NITFAB_0203	CDS	193708	194415		putative DsbA-like protein
Cytochrome c biogenesis	NITFAB_0437	CDS	447234	449078	*dsbD*	Thiol:disulfide interchange protein DsbD
Cytochrome c biogenesis	NITFAB_2312	CDS	2320186	2320662	*ccmE*	heme chaperone
Cytochrome c biogenesis	NITFAB_2313	CDS	2320786	2320950		putative Heme exporter protein CcmD (fragment)
Cytochrome c biogenesis	NITFAB_2314	CDS	2320947	2321624	*ccmC*	heme exporter subunit; membrane component of ABC superfamily
Cytochrome c biogenesis	NITFAB_2315	CDS	2321716	2322399	*ccmB*	heme exporter subunit; membrane component of ABC superfamily
Cytochrome c biogenesis	NITFAB_2316	CDS	2322396	2323010	*ccmA*	heme exporter subunit; ATP-binding component of ABC superfamily
Cytochrome c biogenesis	NITFAB_2519	CDS	2533438	2533950		putative Disulfide bond formation protein B DsbB
Cytochromes	NITFAB_0005	CDS	4899	5273		putative Cytochrome c class I
Cytochromes	NITFAB_0057	CDS	49810	50406		putative monohaem cytochrome c
Cytochromes	NITFAB_0058	CDS	50531	51310		Dihaem cytochrome c
Cytochromes	NITFAB_0187	CDS	175579	176259		Dihaem cytochrome c
Cytochromes	NITFAB_0188	CDS	176249	176599		Monohaem cytochrome c
Cytochromes	NITFAB_0192	CDS	178774	179205		putative Monohaem cytochrome c
Cytochromes	NITFAB_1009	CDS	1042294	1042614		putative Cytochrome c class I
Cytochromes	NITFAB_1049	CDS	1077880	1078497		putative Cytochrome b
Cytochromes	NITFAB_1656	CDS	1670809	1672320		putative Multihaem cytochrome c
Cytochromes	NITFAB_1911	CDS	1906967	1907455	*cybB*	Cytochrome b561

Cytochromes	NITFAB_2103	CDS	2111941	2112516		putative cytochrome c
Cytochromes	NITFAB_2210	CDS	2223161	2223841		putative Cytochrome c-551
Cytochromes	NITFAB_2356	CDS	2360153	2361046		putative Cytochrome c family protein
FeS Cluster Assembly	NITFAB_2525	CDS	2537721	2538206	*sufU*	FeS cluster assembly SUF system, sulfur-transfer protein SufU
FeS Cluster Assembly	NITFAB_2527	CDS	2539462	2540769	*sufD*	FeS cluster assembly SUF system , protein SufD
FeS Cluster Assembly	NITFAB_2528	CDS	2540766	2541557	*sufC*	FeS cluster assembly SUF system, ATPase SufC
FeS Cluster Assembly	NITFAB_2529	CDS	2541554	2542990	*sufB*	FeS cluster assembly SUF system, protein SufB
FeS Cluster Assembly	NITFAB_2530	CDS	2543076	2543435	*sufA*	FeS cluster assembly SUF system, protein SufA
Iron Metabolism	NITFAB_1367	CDS	1404069	1405892		putative siderophore biosynthesis protein
Iron Metabolism	NITFAB_1371	CDS	1408682	1410799		putative TonB-dependent siderophore receptor
Iron Metabolism	NITFAB_1391	CDS	1429456	1429860		putative ferric reductase (fragment)
Iron Metabolism	NITFAB_1392	CDS	1429820	1430251		putative ferric reductase (fragment)
Iron Metabolism	NITFAB_1393	CDS	1430369	1430788		putative Ferric reductase domain (fragment)
Iron Metabolism	NITFAB_1692	CDS	1708850	1709389		putative Bacterioferritin
Iron Metabolism	NITFAB_1876	CDS	1855863	1858343		putative TonB-dependent siderophore receptor
Iron Metabolism	NITFAB_1999	CDS	2005271	2006299		putative Ferric iron ABC transporter, iron-binding protein
Iron Metabolism	NITFAB_2000	CDS	2006309	2007955		putative Iron ABC transporter permease protein
Iron Metabolism	NITFAB_2001	CDS	2008056	2009126		putative Fe(3+)-transporting ATPase
Iron Metabolism	NITFAB_2003	CDS	2009256	2009681	*fur*	DNA-binding transcriptional dual regulator of siderophore biosynthesis and transport
Iron Metabolism	NITFAB_2056	CDS	2066098	2068296		putative TonB-dependent receptor
Iron Metabolism	NITFAB_2086	CDS	2098496	2098960	*bfr*	bacterioferritin, iron storage and detoxification protein
Magnesium Transport	NITFAB_0324	CDS	325549	326814		putative magnesium/nickel/cobalt transporter (fragment)
Magnesium Transport	NITFAB_0408	CDS	411945	414539	*mgtA*	Magnesium-transporting ATPase, P-type 1
Magnesium Transport	NITFAB_2346	CDS	2350309	2351763		putative Magnesium transporter
Mechanosensitive Channel	NITFAB_0994	CDS	1027439	1027843	*mscL*	mechanosensitive channel
Molybdenum Transport	NITFAB_2422	CDS	2430033	2431103	*modC*	Molybdenum import ATP-binding protein ModC
Molybdenum Transport	NITFAB_2423	CDS	2431100	2431804	*modB*	Molybdenum transport system permease protein ModB
Molybdenum Transport	NITFAB_2424	CDS	2431819	2432595		putative Molybdenum ABC transporter, periplasmic molybdenum-binding protein ModA
Monovalent Cation/Proton Antiporter	NITFAB_1283	CDS	1307823	1309199	*nhaA*	Na(+)/H(+) antiporter NhaA
Monovalent Cation/Proton Antiporter	NITFAB_2132	CDS	2146579	2149503	*mrpA B*	monovalent cation/H(+) antiporter subunit A/B
Monovalent Cation/Proton Antiporter	NITFAB_2133	CDS	2149503	2149850	*mrpC*	monovalent cation/H(+) antiporter subunit C
Monovalent Cation/Proton Antiporter	NITFAB_2134	CDS	2149906	2151522	*mrpD*	proton transporter component of monovalent cation/H+ antiporter subunit D
Monovalent Cation/Proton Antiporter	NITFAB_2135	CDS	2151474	2151716		protein of unknown function

Monovalent Cation/Proton Antiporter	NITFAB_2136	CDS	2151519	2152007		putative monovalent cation/H(+) antiporter subunit E
Monovalent Cation/Proton Antiporter	NITFAB_2137	CDS	2152004	2152285	*mrpF*	monovalent cation/H(+) antiporter subunit F
Monovalent Cation/Proton Antiporter	NITFAB_2138	CDS	2152282	2152593	*mrpG*	monovalent cation/H(+) antiporter subunit G
Peroxidase	NITFAB_1212	CDS	1229745	1230830		putative Cytochrome c peroxidase (ccp)
Peroxidase	NITFAB_1373	CDS	1412079	1413359		putative Di-heme cytochrome c peroxidase
Peroxidase	NITFAB_1903	CDS	1893290	1894333	*ccpA*	Cytochrome c551 peroxidase
Phosphate Transport	NITFAB_0743	CDS	765491	766264	*pstB1*	phosphate transporter subunit; ATP-binding component of ABC superfamily
Phosphate Transport	NITFAB_0744	CDS	766277	767122	*pstA1*	phosphate transporter subunit; membrane component of ABC superfamily
Phosphate Transport	NITFAB_0745	CDS	767125	768087	*pstC1*	phosphate transporter subunit; membrane component of ABC superfamily
Phosphate Transport	NITFAB_0746	CDS	768211	769260	*pstS1*	phosphate transporter subunit; periplasmic-binding component of ABC superfamily
Phosphate Transport	NITFAB_0967	CDS	1005708	1006757	*pstS2*	phosphate transporter subunit; periplasmic-binding component of ABC superfamily
Phosphate Transport	NITFAB_1051	CDS	1080264	1081037	*pstB2*	high-affinity phosphate transport protein (ABC superfamily, atp_bind)
Phosphate Transport	NITFAB_1052	CDS	1081050	1081895	*pstA2*	phosphate transporter subunit; membrane component of ABC superfamily
Phosphate Transport	NITFAB_1053	CDS	1081898	1082860	*pstC2*	phosphate transporter subunit; membrane component of ABC superfamily
Phosphate Transport	NITFAB_1054	CDS	1082984	1084033	*pstS3*	phosphate transporter subunit; periplasmic-binding component of ABC superfamily
Phosphate Transport	NITFAB_2482	CDS	2496355	2497062	*phoU*	negative regulator of PhoR/PhoB two-component regulator
Potassium Transport	NITFAB_1894	CDS	1881154	1883025	*kup*	potassium transporter
Quarternary Ammonium Compound resistance	NITFAB_1750	CDS	1754843	1755163	*sugE*	Quaternary ammonium compound-resistance protein SugE
Quarternary Ammonium Compound resistance	NITFAB_p0005	CDS	3780	4112	*emrE*	Quaternary ammonium compound-resistance protein EmrE
Quorum Sensing	NITFAB_1168	CDS	1181820	1182377	*anoI*	Acyl-homoserine-lactone synthase
Quorum Sensing	NITFAB_2191	CDS	2206256	2206843	*anoI*	Acyl-homoserine-lactone synthase
Quorum Sensing	NITFAB_2193	CDS	2207440	2208222	*anoR*	Transcriptional activator protein AnoR
RND family Heavy metal/cation/multidrug Efflux	NITFAB_0193	CDS	179386	180711		putative Cation efflux system, membrane fusion protein
RND family Heavy metal/cation/multidrug Efflux	NITFAB_0194	CDS	180708	183818	*cusA*	Cation efflux system protein CusA
RND family Heavy metal/cation/multidrug Efflux	NITFAB_0537	CDS	535178	536479		putative Outer membrane efflux protein, cusC-like
RND family Heavy metal/cation/multidrug Efflux	NITFAB_0538	CDS	536476	537627		putative Efflux transporter, RND family, MFP subunit
RND family Heavy metal/cation/multidrug Efflux	NITFAB_0539	CDS	537629	540727	*cusA2*	Cation efflux system protein
RND family Heavy metal/cation/multidrug Efflux	NITFAB_1570	CDS	1604686	1607775		putative Cation efflux system protein CzcA

RND family Heavy metal/cation/multidrug Efflux	NITFAB_1571	CDS	1607785	1609026		putative Efflux transporter, RND family, MFP subunit
RND family Heavy metal/cation/multidrug Efflux	NITFAB_1572	CDS	1609028	1610380		putative Outer membrane efflux protein
RND family Heavy metal/cation/multidrug Efflux	NITFAB_1586	CDS	1618626	1621790	*cusA3*	copper/silver efflux system, membrane component
RND family Heavy metal/cation/multidrug Efflux	NITFAB_1587	CDS	1621787	1623397		putative Cation efflux system protein CusB
RND family Heavy metal/cation/multidrug Efflux	NITFAB_1588	CDS	1623394	1624737		putative outer membrane silver resistance, three components proton antiporter efflux system SilC
RND family Heavy metal/cation/multidrug Efflux	NITFAB_1602	CDS	1631892	1633019		putative RND efflux pump, membrane fusion protein, similar to AcrA family
RND family Heavy metal/cation/multidrug Efflux	NITFAB_1603	CDS	1633016	1636099		putative RND efflux pump, similar to multidrug efflux transporter AcrB
RND family Heavy metal/cation/multidrug Efflux	NITFAB_1757	CDS	1760980	1764081		putative Heavy metal efflux pump
RND family Heavy metal/cation/multidrug Efflux	NITFAB_1864	CDS	1847929	1848708		putative Zinc transporter
RND family Heavy metal/cation/multidrug Efflux	NITFAB_1968	CDS	1964135	1965367	*mdtA*	multidrug efflux system, subunit A
RND family Heavy metal/cation/multidrug Efflux	NITFAB_1969	CDS	1965364	1968453	*mdtB*	multidrug efflux system, subunit B
RND family Heavy metal/cation/multidrug Efflux	NITFAB_1970	CDS	1968476	1971583	*mdtC*	multidrug efflux system, subunit C
RND family Heavy metal/cation/multidrug Efflux	NITFAB_2005	CDS	2010740	2012266		putative RND efflux system, outer membrane lipoprotein, NodT family
RND family Heavy metal/cation/multidrug Efflux	NITFAB_2006	CDS	2012244	2015381	*acrB*	multidrug efflux system protein
RND family Heavy metal/cation/multidrug Efflux	NITFAB_2007	CDS	2015390	2016550	*acrE*	RND efflux pump, membrane fusion protein
Sec Pathway	NITFAB_0271	CDS	265750	268467	*secA*	Preprotein translocase subunit SecA, ATPase
Sec Pathway	NITFAB_0463	CDS	471358	471702	*secE*	Preprotein translocase, subunit SecE
Sec Pathway	NITFAB_0497	CDS	497006	498337	*secY*	Preprotein translocase, membrane subunit SecY
Sec Pathway	NITFAB_0943	CDS	984982	985785	*lepB*	leader peptidase (signal peptidase I)
Sec Pathway	NITFAB_1106	CDS	1141882	1142232	*secG*	Preprotein translocase, membrane subunit SecG
Sec Pathway	NITFAB_1253	CDS	1275506	1275961	*lspA*	prolipoprotein signal peptidase (signal peptidase II)
Sec Pathway	NITFAB_1761	CDS	1768203	1769132	*secF*	Protein-export membrane protein SecF
Sec Pathway	NITFAB_1762	CDS	1769168	1770979	*secD*	Protein-export membrane protein SecD
Sec Pathway	NITFAB_1763	CDS	1770989	1771312	*yajC*	Preprotein translocase, subunit YajC
Sec Pathway	NITFAB_2081	CDS	2093522	2094580	*ftsY*	Signal recognition particle receptor FtsY
Sec Pathway	NITFAB_2288	CDS	2300518	2300997	*secB*	protein export chaperone
Sec Pathway	NITFAB_2354	CDS	2356946	2358298	*ffh*	Signal Recognition Particle (SRP) component with 4.5S RNA (ffs)

Superoxide dismutase	NITFAB_0901	CDS	937741	938319	*sodB*	superoxide dismutase (Fe)
Superoxide dismutase	NITFAB_1655	CDS	1670177	1670785		putative superoxide dismutase (copper/zinc)
TAT secretion	NITFAB_2544	CDS	2555372	2556127	*tatC*	Sec-independent protein translocase protein TatC
TAT secretion	NITFAB_2545	CDS	2556127	2556855		putative Sec-independent protein translocase protein TatB
TAT secretion	NITFAB_2546	CDS	2556871	2557101	*tatA*	Sec-independent protein translocase protein TatA
Toluene transport (export)	NITFAB_0135	CDS	119077	119886	*yrbF*	toluene transporter subunit: ATP-binding component of ABC superfamily
Toluene transport (export)	NITFAB_0136	CDS	119877	120671	*yrbE*	toluene transporter subunit: membrane component of ABC superfamily
Toluene transport (export)	NITFAB_0137	CDS	120674	121135	*yrbD*	toluene transporter subunit: membrane component of ABC superfamily
Toluene transport (export)	NITFAB_0138	CDS	121167	121808		putative ABC-type toluene transporter, auxiliary component
Toluene transport (export)	NITFAB_0140	CDS	122124	123029	*yadG*	putative transporter subunit: ATP-binding component of ABC superfamily
Toluene transport (export)	NITFAB_0141	CDS	123037	123792		putative transporter subunit: membrane component of ABC superfamily
Ubiquinone Synthesis	NITFAB_0124	CDS	108342	109499	*ubiF*	Ubiquinone biosynthesis hydroxylase, UbiH/UbiF/VisC/COQ6 family
Ubiquinone Synthesis	NITFAB_0128	CDS	112688	114151	*ubiD*	3-octaprenyl-4-hydroxybenzoate decarboxylase
Ubiquinone Synthesis	NITFAB_0233	CDS	225742	226899	*ubiH*	2-octaprenyl-6-methoxyphenol hydroxylase, FAD/NAD(P)-binding
Ubiquinone Synthesis	NITFAB_0325	CDS	326861	327718	*ubiA*	p-hydroxybenzoate octaprenyltransferase
Ubiquinone Synthesis	NITFAB_0326	CDS	327715	328335	*ubiC*	putative chorismate pyruvate-lyase ubiC
Ubiquinone Synthesis	NITFAB_0412	CDS	419530	421050	*ubiB*	2-octaprenylphenol hydroxylase
Ubiquinone Synthesis	NITFAB_0415	CDS	422717	423451	*ubiE*	bifunctional 2-octaprenyl-6-methoxy-1,4-benzoquinone methylase and S-adenosylmethionine:2-DMK methyltransferase
Ubiquinone Synthesis	NITFAB_0626	CDS	619536	620231	*ubiG*	3-demethylubiquinone-9 3-O-methyltransferase and 2-octaprenyl-6-hydroxy phenol methylase
**Nitrogen Metabolism**						
**Pathway**	**Label**	**Type**	**Begin**	**End**	**Gene**	**Product**
Ammonium Transporter	NITFAB_1755	CDS	1758819	1760042		putative Ammonium transporter
Nitrite Assimilation	NITFAB_1038	CDS	1067122	1069557	*nasD*	Assimilatory Nitrite reductase, NAD(P)H dependent, large subunit
Nitrite Assimilation	NITFAB_1039	CDS	1069570	1069893	*nasE*	Assimilatory Nitrite reductase, NAD(P)H dependent, small subunit
Nitrite Oxidation	NITFAB_2341	CDS	2341774	2342514		putative nitrite oxidoreductase assembly chaperone protein
Nitrite Oxidation	NITFAB_2342	CDS	2342583	2343692		putative nitrite oxidoreductase, gamma subunit
Nitrite Oxidation	NITFAB_2343	CDS	2343703	2344860	*nxrB1*	Nitrite oxidoreductase, beta subunit
Nitrite Oxidation	NITFAB_2344	CDS	2344941	2348450	*nxrA1*	Nitrite oxidoreductase, alpha subunit
Nitrite Oxidation	NITFAB_2593	CDS	2594871	2595611		putative nitrite oxidoreductase assembly chaperone protein
Nitrite Oxidation	NITFAB_2594	CDS	2595680	2596789		putative nitrite oxidoreductase, gamma subunit
Nitrite Oxidation	NITFAB_2595	CDS	2596800	2597957	*nxrB2*	Nitrite oxidoreductase, beta subunit
Nitrite Oxidation	NITFAB_2596	CDS	2598038	2601547	*nxrA2*	Nitrite oxidoreductase, alpha subunit
Nitrite Reduction	NITFAB_0189	CDS	176679	177623	*nirK*	Copper-containing nitrite reductase (NO-forming)
Nitrite Transporter	NITFAB_1230	CDS	1246759	1247571		putative Formate/nitrite transporter
**Respiratory Chain**						
**Pathway**	**Label**	**Type**	**Begin**	**End**	**Gene**	**Product**

Alternative Complex III (ACIII)	NITFAB_0001	CDS	356	2566		putative Menaquinol oxidoreductase complex ACIII, molybdopterin-binding subunit ActB1
Alternative Complex III (ACIII)	NITFAB_0002	CDS	2610	3215		putative Menaquinol oxidoreductase complex ACIII, cytochrome c subunit ActA
Alternative Complex III (ACIII)	NITFAB_2604	CDS	2605395	2606408		putative Menaquinol oxidoreductase complex ACIII, membrane subunit ActF
Alternative Complex III (ACIII)	NITFAB_2605	CDS	2606422	2606961		putative Menaquinol oxidoreductase complex ACIII, monohaem cytochrome c subunit ActE
Alternative Complex III (ACIII)	NITFAB_2606	CDS	2606958	2607485		putative Menaquinol oxidoreductase complex ACIII, DUF3341 subunit ActD
Alternative Complex III (ACIII)	NITFAB_2607	CDS	2607485	2608849		putative Menaquinol oxidoreductase complex ACIII, menaquinol-binding membrane protein subunit ActC
Alternative Complex III (ACIII)	NITFAB_2608	CDS	2608924	2609424		putative Menaquinol oxidoreductase complex ACIII, iron-sulfur cluster-binding subunit ActB2 (C-terminal fragment)
Alternative Complex III (ACIII)	NITFAB_CDS2R	CDS	2	262		putative Menaquinol oxidoreductase complex ACIII, iron-sulfur cluster-binding subunit ActB2 (N-terminal fragment)
Complex I	NITFAB_1772	CDS	1777637	1779088	*nuoN*	NADH-quinone oxidoreductase subunit N
Complex I	NITFAB_1773	CDS	1779111	1780595	*nuoM*	NADH-quinone oxidoreductase subunit M
Complex I	NITFAB_1774	CDS	1780626	1782647	*nuoL*	NADH-quinone oxidoreductase subunit L
Complex I	NITFAB_1775	CDS	1782654	1782965	*nuoK*	NADH-quinone oxidoreductase subunit K
Complex I	NITFAB_1776	CDS	1783114	1783734	*nuoJ*	NADH-quinone oxidoreductase subunit J
Complex I	NITFAB_1777	CDS	1783902	1784390	*nuoI*	NADH-quinone oxidoreductase subunit I
Complex I	NITFAB_1778	CDS	1784410	1785450	*nuoH*	NADH:ubiquinone oxidoreductase, subunit H
Complex I	NITFAB_1779	CDS	1785447	1787825	*nuoG*	NADH-quinone oxidoreductase, subunit G
Complex I	NITFAB_1780	CDS	1787840	1789111	*nuoF*	NADH:ubiquinone oxidoreductase, subunit F
Complex I	NITFAB_1781	CDS	1789108	1789587	*nuoE*	NADH-quinone oxidoreductase, subunit E
Complex I	NITFAB_1782	CDS	1789695	1790948	*nuoD*	NADH-quinone oxidoreductase subunit D
Complex I	NITFAB_1783	CDS	1791054	1791695	*nuoC*	NADH-quinone oxidoreductase subunit C
Complex I	NITFAB_1784	CDS	1791751	1792227	*nuoB*	NADH-quinone oxidoreductase subunit B
Complex I	NITFAB_1785	CDS	1792218	1792574	*nuoA*	NADH-quinone oxidoreductase subunit A
Complex II	NITFAB_0684	CDS	698060	698434	*sdhC*	Succinate dehydrogenase, cytochrome b556 subunit
Complex II	NITFAB_0685	CDS	698428	698802	*sdhD*	Succinate dehydrogenase, hydrophobic membrane anchor protein
Complex II	NITFAB_0686	CDS	698802	700565	*sdhA*	Succinate dehydrogenase, flavoprotein subunit
Complex II	NITFAB_0687	CDS	700925	701620	*sdhB*	Succinate dehydrogenase, iron-sulfur subunit
Complex IV	NITFAB_0046	CDS	42673	43638	*ccoO*	Cbb3-type cytochrome oxidase, cytochrome c subunit
Complex IV	NITFAB_0047	CDS	43674	45083	*ccoN*	Cbb3-type cytochrome oxidase, subunit I
Complex IV	NITFAB_0056	CDS	49317	49829		putative Cbb3-type cytochrome oxidase-like protein
Complex V	NITFAB_0146	CDS	128827	129255	*atpC*	F1 sector of membrane-bound ATP synthase, epsilon subunit
Complex V	NITFAB_0147	CDS	129304	130725	*atpD*	F1 sector of membrane-bound ATP synthase, beta subunit
Complex V	NITFAB_0148	CDS	130927	131796	*atpG*	F1 sector of membrane-bound ATP synthase, gamma subunit
Complex V	NITFAB_0149	CDS	131812	133353	*atpA*	F1 sector of membrane-bound ATP synthase, alpha subunit
Complex V	NITFAB_0150	CDS	133385	133918	*atpH*	F1 sector of membrane-bound ATP synthase, delta subunit

Complex V	NITFAB_0151	CDS	133924	134394	*atpF*	F0 sector of membrane-bound ATP synthase, subunit b
Complex V	NITFAB_0152	CDS	134429	134671	*atpE*	F0 sector of membrane-bound ATP synthase, subunit c
Complex V	NITFAB_0153	CDS	134730	135590	*atpB*	F0 sector of membrane-bound ATP synthase, subunit a
Complex V	NITFAB_0154	CDS	135587	135952		putative ATP synthase, subunit I
**RNAs**						
**Pathway**	**Label**	**Type**	**Begin**	**End**	**Gene**	**Product**
rRNA	NITFAB_16s_rRNA_1	rRNA	2414812	2416339		ribosomal RNA, 16s_rRNA
rRNA	NITFAB_16s_rRNA_2	rRNA	2002980	2004507		ribosomal RNA, 16s_rRNA
rRNA	NITFAB_23s_rRNA_1	rRNA	2411340	2414224		ribosomal RNA, 23s_rRNA
rRNA	NITFAB_23s_rRNA_2	rRNA	1999508	2002392		ribosomal RNA, 23s_rRNA
rRNA	NITFAB_5s_rRNA_1	rRNA	2411105	2411216		ribosomal RNA, 5s_rRNA
rRNA	NITFAB_5s_rRNA_2	rRNA	1999273	1999384		ribosomal RNA, 5s_rRNA
tRNA	NITFAB_tmRNA6	tmRNA	1843652	1844020		tmRNA
tRNA	NITFAB_tRNA1	tRNA	51890	51966		Arg tRNA
tRNA	NITFAB_tRNA10	tRNA	602524	602600		Met tRNA
tRNA	NITFAB_tRNA11	tRNA	709368	709452		Leu tRNA
tRNA	NITFAB_tRNA12	tRNA	715552	715627		Val tRNA
tRNA	NITFAB_tRNA13	tRNA	715639	715715		Asp tRNA
tRNA	NITFAB_tRNA14	tRNA	791095	791170		Arg tRNA
tRNA	NITFAB_tRNA15	tRNA	946270	946359		Ser tRNA
tRNA	NITFAB_tRNA16	tRNA	1142253	1142337		Leu tRNA
tRNA	NITFAB_tRNA17	tRNA	1331693	1331785		Ser tRNA
tRNA	NITFAB_tRNA18	tRNA	1331812	1331888		Arg tRNA
tRNA	NITFAB_tRNA19	tRNA	1448189	1448264		Gly tRNA
tRNA	NITFAB_tRNA2	tRNA	375633	375708		Phe tRNA
tRNA	NITFAB_tRNA20	tRNA	1448322	1448395		Cys tRNA
tRNA	NITFAB_tRNA21	tRNA	1448457	1448533		Pro tRNA
tRNA	NITFAB_tRNA22	tRNA	1491971	1492046		Lys tRNA
tRNA	NITFAB_tRNA23	tRNA	1740015	1740103		Leu tRNA
tRNA	NITFAB_tRNA24	tRNA	1753309	1753393		Leu tRNA
tRNA	NITFAB_tRNA25	tRNA	1912389	1912464		Ala tRNA
tRNA	NITFAB_tRNA26	tRNA	1912488	1912563		Glu tRNA
tRNA	NITFAB_tRNA27	tRNA	2589815	2589890		Thr tRNA

tRNA	NITFAB_tRNA28	tRNA	2414608	2414684		Ile tRNA
tRNA	NITFAB_tRNA29	tRNA	2414527	2414602		Ala tRNA
tRNA	NITFAB_tRNA3	tRNA	469563	469647		Tyr tRNA
tRNA	NITFAB_tRNA30	tRNA	2410795	2410881		Leu tRNA
tRNA	NITFAB_tRNA31	tRNA	2279241	2279332		Ser tRNA
tRNA	NITFAB_tRNA32	tRNA	2119279	2119369		Ser tRNA
tRNA	NITFAB_tRNA33	tRNA	2004741	2004817		Met tRNA
tRNA	NITFAB_tRNA34	tRNA	2002776	2002852		Ile tRNA
tRNA	NITFAB_tRNA35	tRNA	2002695	2002770		Ala tRNA
tRNA	NITFAB_tRNA36	tRNA	1910664	1910740		Val tRNA
tRNA	NITFAB_tRNA37	tRNA	1663771	1663847		Pro tRNA
tRNA	NITFAB_tRNA38	tRNA	1578123	1578199		Arg tRNA
tRNA	NITFAB_tRNA39	tRNA	1578018	1578093		His tRNA
tRNA	NITFAB_tRNA4	tRNA	469756	469829		Gly tRNA
tRNA	NITFAB_tRNA40	tRNA	1125124	1125199		Asn tRNA
tRNA	NITFAB_tRNA41	tRNA	307277	307353		Met tRNA
tRNA	NITFAB_tRNA42	tRNA	207082	207155		Gly tRNA
tRNA	NITFAB_tRNA5	tRNA	469840	469914		Thr tRNA
tRNA	NITFAB_tRNA6	tRNA	471249	471324		Trp tRNA
tRNA	NITFAB_tRNA7	tRNA	548995	549069		Gln tRNA
tRNA	NITFAB_tRNA8	tRNA	570123	570198		Lys tRNA
tRNA	NITFAB_tRNA9	tRNA	570257	570332		Thr tRNA
**Sulfur Metabolism**						
**Pathway**	**Label**	**Type**	**Begin**	**End**	**Gene**	**Product**
Sulfate Import	NITFAB_1675	CDS	1693177	1694232	*cysA*	sulfate/thiosulfate import ATP-binding protein CysA
Sulfate Import	NITFAB_1676	CDS	1694229	1695110	*cysW*	sulfate import protein (ABC superfamily, membrane subunit)
Sulfate Import	NITFAB_1677	CDS	1695107	1695937	*cysT*	sulfate transport protein (ABC superfamily, membrane subunit)
Sulfate Import	NITFAB_1685	CDS	1699887	1700900	*sbp*	sulfate transporter subunit; periplasmic-binding component of ABC superfamily
Sulfur Assimilation	NITFAB_0850	CDS	880756	882501	*sir*	assimilatory sulfite reductase (ferredoxin)
Sulfur Assimilation	NITFAB_0852	CDS	883006	883731	*cysH*	adenylylsulfate reductase, thioredoxin dependent
Sulfur Assimilation	NITFAB_0854	CDS	883841	884746	*cysD*	sulfate adenylyltransferase, subunit 2
Sulfur Assimilation	NITFAB_0855	CDS	884749	886002	*cysN*	sulfate adenylyltransferase, subunit 1
Sulfur Assimilation	NITFAB_2037	CDS	2042834	2043718	*cysK1*	cysteine synthase A, O-acetylserine sulfhydrolase A subunit
Sulfur Assimilation	NITFAB_2043	CDS	2047067	2047954	*cysM*	cysteine synthase B, O-acetylserine sulfhydrolase B subunit
Sulfur Assimilation	NITFAB_2345	CDS	2348995	2349924	*cysK2*	cysteine synthase A, O-acetylserine sulfhydrolase A subunit
Sulfite Oxidoreductase	NITFAB_0978	CDS	1012389	1013624	*sorA*	sulfite:cytochrome c oxidoreductase, subunit A
Sulfite Oxidoreductase	NITFAB_0979	CDS	1013634	1014266	*sorB*	sulfite:cytochrome c oxidoreductase, subunit B
**Prophage**						

Pathway	Label	Type	Begin	End	Gene	Product
Predicted functional Prophage	NITFAB_1795	CDS	1796778	1798265		protein of unknown function
Predicted functional Prophage	NITFAB_1796	CDS	1798344	1799444		protein of unknown function
Predicted functional Prophage	NITFAB_1799	CDS	1800230	1802245		membrane protein of unknown function
Predicted functional Prophage	NITFAB_1800	CDS	1802289	1802540		conserved protein of unknown function
Predicted functional Prophage	NITFAB_1801	CDS	1802609	1802917		conserved protein of unknown function
Predicted functional Prophage	NITFAB_1802	CDS	1803010	1803666		putative phage tail protein
Predicted functional Prophage	NITFAB_1806	CDS	1804599	1805543		conserved protein of unknown function
Predicted functional Prophage	NITFAB_1807	CDS	1805555	1805893		putative Phage-related exported protein
Predicted functional Prophage	NITFAB_1808	CDS	1805895	1807187		ClpP class periplasmic serine protease
Predicted functional Prophage	NITFAB_1809	CDS	1807184	1808887		Phage portal protein, lambda family
Predicted functional Prophage	NITFAB_1810	CDS	1808841	1809068		conserved protein of unknown function
Predicted functional Prophage	NITFAB_1811	CDS	1809206	1809511		conserved protein of unknown function
Predicted functional Prophage	NITFAB_1812	CDS	1809504	1809791		conserved protein of unknown function
Predicted functional Prophage	NITFAB_1817	CDS	1811974	1813905		Phage terminase GpA
Predicted functional Prophage	NITFAB_1818	CDS	1813871	1814251		protein of unknown function
Predicted functional Prophage	NITFAB_1820	CDS	1814617	1815180		protein of unknown function
Predicted functional Prophage	NITFAB_1824	CDS	1816266	1816547		conserved membrane protein of unknown function

Predicted functional Prophage	NITFAB_1825	CDS	1816544	1817140		protein of unknown function
Predicted functional Prophage	NITFAB_1827	CDS	1817688	1819391		exported protein of unknown function
Predicted functional Prophage	NITFAB_1832	CDS	1823121	1823621		Uncharacterized endonuclease
Predicted functional Prophage	NITFAB_1835	CDS	1824390	1825679		DNA modification methylase
Predicted functional Prophage	NITFAB_1838	CDS	1826613	1826876		conserved protein of unknown function
Predicted functional Prophage	NITFAB_1840	CDS	1827241	1827798		protein of unknown function
Predicted functional Prophage	NITFAB_1843	CDS	1828936	1831212		DNA primase/helicase, phage-associated
Predicted functional Prophage	NITFAB_1845	CDS	1831477	1831788		conserved protein of unknown function
Predicted functional Prophage	NITFAB_1846	CDS	1832045	1832278		conserved protein of unknown function
Predicted functional Prophage	NITFAB_1848	CDS	1832959	1833237		conserved protein of unknown function
