# Supplementary material for: Characterization of the First “Candidatus Nitrotoga” Isolate Reveals Metabolic Versatility and Separate Evolution of Widespread Nitrite-Oxidizing Bacteria
Source: mBio. 2018 Jul 10;9(4):e01186-18. doi: 10.1128/mBio.01186-18 (PMC6050957; doi:10.1128/mBio.01186-18)
Supplement: TEXT S1 [file mbo004183968s1.pdf]

Supplemental Text S1: Supplemental materials and methods

Electron microscopy

Scanning electron microscopy was performed as described previously (1) with the following modifications. Sterile poly-L-lysine coated slides were submerged in an actively growing "*Ca*. N. fabula" culture for 3 days before fixation of attached cells. All fixatives were diluted in, and all washing steps were performed with, cacodylate buffer (25 mM sodium cacodylate, 0.7 mM $MgCl_2$, pH 7.0) mimicking medium osmolarity. For transmission electron microscopy following chemical fixation, 300 ml of a late exponential phase "*Ca*. N. fabula" culture was fixed by adding glutaraldehyde (2.5% vol/vol final concentration) and harvested by centrifugation (9,000×g, 15 min). Cells were embedded in 1% (wt/vol) plaque agarose (Biozym) in cacodylate buffer, cut into 1 mm-sized blocks and post-fixed with a 1% (wt/vol) OsO_4 solution in cacodylate buffer for 1 h. Fixed cells were washed three times in cacodylate buffer, dehydrated in 30 to 100% (vol/vol) ethanol, washed twice in propylene oxide, and infiltrated with increasing concentrations of low viscosity resin in propylene oxide. For polymerization, the resin blocks were incubated at 60°C for one week. Ultra-thin sections (70 nm) were cut from the resin blocks (Ultracut S, Leica) with a glass knife. Sections were placed on copper grid mesh holders and post-stained with gadolinium triacetate and lead citrate before visualization with a Libra120 transmission electron microscope (Zeiss). For transmission electron microscopy following high pressure freezing, concentrated live culture was mixed with 2% agarose in a 3 mm aluminum sample holder and immediately high-pressure frozen with an HPM 100 (Leica). Samples were transferred onto frozen acetone containing 1% (wt/vol) OsO_4 and processed using the super quick freeze-substitution method (2). After reaching room temperature, the samples were washed three times with acetone and two times with ethanol, and were infiltrated sequentially using centrifugation (3) in 2 ml tubes

with 25, 50, 75 and 2× 100% LR-White resin (Agar Scientific). The samples were placed on top of the resin and centrifuged for 30 s at 2,000×g in each step. After the second pure resin step, samples were transferred into fresh resin in gelatin capsules and polymerized at 60°C for 1 h (4). Ultra-thin (70 nm) sections were cut with an Ultracut UC7 (Leica) and mounted on formvar coated slot grids (Agar Scientific). Sections were contrasted with 0.5% aqueous uranyl acetate (Science Services) for 20 min and with 2% Reynold's lead citrate for 6 min before imaging with a Quanta FEG 250 scanning electron microscope (FEI) equipped with a STEM detector.

Nitrite oxidation kinetic measurements

Nitrite oxidation kinetics of "*Ca*. N. fabula" were inferred from instantaneous oxygen uptake measurements in four independent experiments as previously described (5). Biomass of "*Ca*. N. fabula" was sampled upon substrate depletion (early stationary phase), which was predictable to 2-3 hours. Oxygen uptake measurements were done using a microrespiration (MR) system submerged in a recirculating water bath (28°C) (6, 7). All measurements were performed utilizing 2 ml glass MR chambers equipped with an MR injection lid, a glass coated stir bar, a PA 2000 picoammeter, and an OX-MR oxygen microsensor with a 500 µm tip diameter (Unisense). Before the experiments, the OX-MR microsensor was polarized continuously for at least one week (6, 7). Culture biomass, either concentrated (10× by centrifugation at 6000×g, 5 min, 20°C) or un-concentrated, was incubated for a minimum of 30 min in the recirculating water bath before transfer to an MR chamber. MR chambers with glass coated stir bars were filled headspace-free with "*Ca*. N. fabula" culture. Once immersed in the recirculating water bath, stirring (350 r.p.m.) was started. The OX-MR microsensor was inserted into the MR chamber and equilibrated (1 to 2 h). Stable sensor signal drift was

measured for at least 10 min prior the initial injection of nitrite using Hamilton syringes. Multiple nitrite injection oxygen uptake measurement traces were performed. Nitrite additions started from low concentrations and moved toward high concentrations (the injections led to different start concentrations of nitrite in the MR chambers). Once the nitrite oxidation rate was stable for 2-5 minutes, another injection was performed. The rate of oxygen uptake was measured after each individual injection of nitrite (5). The endogenous rate of oxygen consumption was subtracted from the measured rates at the different nitrite concentrations. Dissolved oxygen (DO) was not a limiting factor in the experiments. The DO concentration in the MR chambers was 200-220 µM at the beginning and 15-160 µM at the end of the experiments. After the experiments, MR chamber contents were immediately frozen for chemical and protein analysis. Nitrite and nitrate concentrations were measured to confirm the total injected nitrite concentration and oxidation to nitrate. Total protein content per MR chamber was determined using the Pierce bicinchoninic acid (BCA) Protein Assay Kit (Thermo Scientific) "Enhanced Test-tube Procedure" after cell lysis (Bacterial Protein Extraction Reagent, Thermo Scientific).

The kinetic constants $K_{m(app)}$ and V_{max} of "*Ca*. N. fabula" were estimated from multiple nitrite injection oxygen uptake measurements. Nitrite uptake rates were calculated from the measured oxygen uptake rates, according to a nitrite to oxygen uptake stoichiometry of 2:1 for NOB. Michaelis-Menten plots of nitrite uptake rates versus nitrite concentration were obtained by fitting a Michaelis-Menten model to the data. A nonlinear least squares regression analysis was used to estimate both $K_{m(app)}$ and V_{max} of "*Ca*. N. fabula" (8).

"*Ca*. N. fabula" genome sequencing and assembly

Illumina sequencing libraries were prepared using the Nextera DNA library prep kit (Illumina

Inc.) following the manufacturer's recommendations and paired-end sequenced (2×300 bp) on a MiSeq using a MiSeq Reagent kit v3 (Illumina) following the manufacturer's recommendations. In addition, Nanopore library preparation was done using the Nanopore sequencing kit (SQK-MAP006, Oxford Nanopore), following the manufacturers recommendations (v. MN006_1124_revF_14Aug2015). The library was sequenced using the MinION Mk1 device (Oxford Nanopore) with the MinKnow software (v. 0.50.2.15). Base calling was carried out using Metrichor and the 2D base calling workflow (Rev. 1.62). Illumina read quality and adaptor trimming (trim limit: 0.01, no ambiguous bases, min length: 55 bp), *de novo* assembly (word size: 21, bubble size: 186, min length: 500 bp), and read mapping (default settings except length fraction: 0.95 and similarity fraction: 0.95) were performed in CLC Genomics Workbench v. 8.5.1. The Illumina *de novo* assembly was checked for contamination and completeness using the mmgenome workflow (http://madsalbertsen.github.io/mmgenome/). Afterwards, the Illumina *de novo* assembly was manually scaffolded with nanopore data (mapping reads to scaffolds ends). Gaps were polished by recruiting Illumina reads mapping to the nanopore reads and performing local gap reassembly with the Illumina reads (read mapping and *de novo* assembly settings were the same as above except read similarity fraction: 0.85).

Enrichment and metagenomic analysis of thermophilic nitrifiers

Biofilm was sampled from a hot spring (77°C, pH ~6) in Grændalur valley, Iceland (N 64° 2' 0" W 21°11'43"). To enrich nitrifying organisms, approximately 0.1 g of the biofilm sample was added to 40 ml sterile mineral medium, which had been prepared according to Koch *et al.* (9). The medium was modified by the addition of 3 µM $Na_2WO_4 \times 2H_2O$ (instead of Na_2MoO_4, which was not added), 3.4 nM $Na_2SeO_3 \times 5H_2O$, and 0.5 mM filter-sterilized NH_4Cl. The

culture was incubated at 75°C and without agitation in 100 ml glass bottles in darkness. The ammonium, nitrite, and nitrate content was checked weekly by using Nessler's reagent (Sigma–Aldrich) and nitrite/nitrate test stripes (Merckoquant, Merck). Ammonium (0.5 mM NH_4Cl) was replenished when completely consumed. The pH was monitored with pH test stripes (Macherey-Nagel) and kept between 6 and 7 by titration with $NaHCO_3$. Aliquots of the ammonia- and nitrite-oxidizing enrichment were transferred into bottles containing fresh medium when 5 to 8 mM of ammonium had been consumed. By this approach, several sub-cultures were established from the same primary enrichment.

For the extraction of genomic DNA, biomass from several bottles was pooled, collected by centrifugation (4,500×g, 20 min, 20°C), and stored at -20°C until further processing. The biomass was then freeze-thawed three times before total nucleic acids were extracted by bead beating for 40 s with speed setting 6.0 in the presence of phosphate buffer, 10% (w/v) SDS, and phenol as described elsewhere (10). Between the addition of phosphate buffer and the addition of SDS and phenol, a 30 min incubation with 2 µl of proteinase K (10 mg/ml) at 37°C and shaking (200 r.p.m.) was added. In total 41.34 ng of DNA was sheared by ultrasonication for 40 s, using Covaris SonoLite v. 2.07.

Sequencing was performed at the next generation sequencing unit of the Vienna Biocenter Core Facilities (www.vbcf.ac.at) using an Illumina HiSeq 2500 instrument to generate paired-end (2×125 bp) reads. Paired end reads were 3' end-trimmed using a q-score of 15 and minimum length of 50 nucleotides. Quality-trimmed reads were assembled using Metaspades v. 3.11.1 (11). Assembly coverage was determined by mapping quality-trimmed reads with bwa v. 0.7.16a (12). Metabat v. 2.12.1 (13) was used to bin metagenome-assembled genomes (MAGs) using tetranucleotide frequency and coverage. CheckM v. 1.0.7 (14) was used to assess MAG completeness and contamination. MAGs were automatically annotated by using an in-house modified version of prokka (15), which uses a local copy of the NCBI non-

redundant protein database (NCBI nr) for blast searches and reports for each predicted gene product the best blast hit, sequence identity to this hit, and query and subject alignment coverage values.

Annotation of *nxr* genes

The *nxr* genes of "*Ca*. N. fabula", and the putative *nxr* genes of the *Desulfurococcaceae*-related crenarchaeon, were identified (i) by screening the automated annotations for predicted *nxr*/*nar*-like genes, and (ii) by blast searching the genomic datasets for members of the type II DMSO reductase family with similarity to known NXR and NAR alpha, beta, and gamma subunits. Phylogenetic analysis (Fig. 6 in the main text) confirmed the affiliation of the predicted alpha subunits with (putative) NARs of nitrate-reducing organisms. In the case of "*Ca*. N. fabula", a detailed sequence comparison to the validated NXR of *Nitrospira defluvii* confirmed the presence of the conserved, cofactor-binding residues in the alpha and beta subunits (see main text). The identified *nxr*/*nar*-like genes were annotated as *nxr* ("*Ca*. N. fabula") or putative *nxr* (*Desulfurococcaceae*-related crenarchaeon) based on the nitrite-oxidizing phenotype of the "*Ca*. N. fabula" isolate and of the nitrifying thermophilic enrichment culture, respectively.

References for Text S1

1. Daebeler A, Herbold CW, Vierheilig J, Sedlacek CJ, Pjevac P, Albertsen M, Kirkegaard RH, de la Torre JR, Daims H, Wagner M. 2018. Cultivation and genomic analysis of "*Candidatus* Nitrosocaldus islandicus," an obligately thermophilic, ammonia-oxidizing Thaumarchaeon from a hot spring biofilm in Graendalur Valley,

Iceland. Front Microbiol 9:193.

2. McDonald KL, Webb RI. 2011. Freeze substitution in 3 hours or less. J Microsc 243:227-233.

3. McDonald KL. 2014. Rapid embedding methods into epoxy and LR White resins for morphological and immunological analysis of cryofixed biological specimens. Microsc Microanal 20:152-163.

4. Bowling AJ, Vaughn KC. 2008. A simple technique to minimize heat damage to specimens during thermal polymerization of LR White in plastic and gelatin capsules. J Microsc 231:186-189.

5. Kits KD, Sedlacek CJ, Lebedeva EV, Han P, Bulaev A, Pjevac P, Daebeler A, Romano S, Albertsen M, Stein LY, Daims H, Wagner M. 2017. Kinetic analysis of a complete nitrifier reveals an oligotrophic lifestyle. Nature 549:269-272.

6. Martens-Habbena W, Berube PM, Urakawa H, de la Torre JR, Stahl DA. 2009. Ammonia oxidation kinetics determine niche separation of nitrifying *Archaea* and *Bacteria*. Nature 461:976-979.

7. Martens-Habbena W, Stahl DA. 2011. Nitrogen metabolism and kinetics of ammonia-oxidizing archaea, p 465-487. *In* Klotz MG, Stein LY (ed), Methods Enzymol, vol 496. Elsevier Academic Press, San Diego.

8. Kemmer G, Keller S. 2010. Nonlinear least-squares data fitting in Excel spreadsheets. Nat Protoc 5:267-281.

9. Koch H, Lücker S, Albertsen M, Kitzinger K, Herbold C, Spieck E, Nielsen PH, Wagner M, Daims H. 2015. Expanded metabolic versatility of ubiquitous nitrite-oxidizing bacteria from the genus *Nitrospira*. Proc Natl Acad Sci U S A 112:11371-11376.

10. Angel R, Claus P, Conrad R. 2012. Methanogenic archaea are globally ubiquitous in aerated soils and become active under wet anoxic conditions. ISME J 6:847-862.

11. Nurk S, Meleshko D, Korobeynikov A, Pevzner PA. 2017. metaSPAdes: a new versatile metagenomic assembler. Genome Res 27:824-834.

12. Li H, Durbin R. 2009. Fast and accurate short read alignment with Burrows-Wheeler

transform. Bioinformatics 25:1754-1760.

13. Kang DWD, Froula J, Egan R, Wang Z. 2015. MetaBAT, an efficient tool for accurately reconstructing single genomes from complex microbial communities. PeerJ 3:e1165.

14. Parks DH, Imelfort M, Skennerton CT, Hugenholtz P, Tyson GW. 2015. CheckM: assessing the quality of microbial genomes recovered from isolates, single cells, and metagenomes. Genome Res 25:1043-1055.

15. Seemann T. 2014. Prokka: rapid prokaryotic genome annotation. Bioinformatics 30:2068-2069.
